# Long-Term Opioid and Gabapentinoid Related Harms in CKD

**DOI:** 10.1016/j.ekir.2026.107085

**Published:** 2026-09-17

**Authors:** Christopher H. Grant, Shona Livingstone, Huan Wang, Karen N. Barnett, Patrick B. Mark, Lesley A. Colvin, Samira Bell

**Affiliations:** 1 Division of Population Health and Genomics, School of Medicine, University of Dundee, Dundee, UK; 2 School of Cardiovascular and Metabolic Health, University of Glasgow, Glasgow, UK; 3 Division of Population and Behavioral Sciences, School of Medicine, University of St Andrews, St Andrews, UK; 4 School of Public Health and Emergency Management, School of Medicine, Southern University of Science and Technology, Shenzhen, China

**Keywords:** chronic kidney disease, chronic pain, gabapentinoids, opioids, pharmacoepidemiology

## Abstract

**Introduction:**

Chronic pain affects over half of the patients with chronic kidney disease (CKD). This study aimed to evaluate the association between new long-term opioids or gabapentinoids and the risk of death, hospitalization, and hip fractures in adults with CKD.

**Methods:**

This is a retrospective, time-updated, population-based cohort study from the East of Scotland (*N* = 953,542) using linked routinely collected data. The study population included adults with biochemically confirmed CKD (estimated glomerular filtration rate [eGFR] <60 ml/min per 1.73 m^2^) without kidney failure from 2010 to 2023. The primary exposures were new, long-term cumulative opioids or gabapentinoids. Discrete time logistic regression modelling with generalized estimating equations and adjustment for relevant covariates and treatment allocation bias was used to determine the association between chronic analgesic use and all-cause mortality.

**Results:**

A total of 30,674 adults with CKD entered the study. Of them, 21,440 patients without prevalent analgesic use were included in the primary and secondary analyses (median follow-up 4.3 years). Among 2399 patients with at least 1 continuous 90-day episode of opioid prescriptions during follow-up, 1159 died. The risk of death increased as continuous 90-day opioid stretches (reference 0) increased from 1 (absolute risk of death: 0.4% [0.3–0.4]; odds ratio [OR]: 1.09 [1.06–1.12], *P* < 0.001) to 6 (absolute risk of death: 0.6% [0.5–0.7]; OR: 1.68 [1.41–2.00], *P* < 0.001). Long-term gabapentinoids were potentially associated with a modest increase in death amongst males (OR: 1.04 [1.00–1.08], *P* = 0.050), but not females (OR: 1.02 [0.99–1.05], *P* = 0.201).

**Conclusion:**

Long-term opioid use is associated with increased mortality and hospitalization in adults with CKD independent of demographics, comorbidities, and treatment allocation bias. These findings support prioritization of safer pain management strategies in people with CKD.

## Graphical abstract

**Figure:**
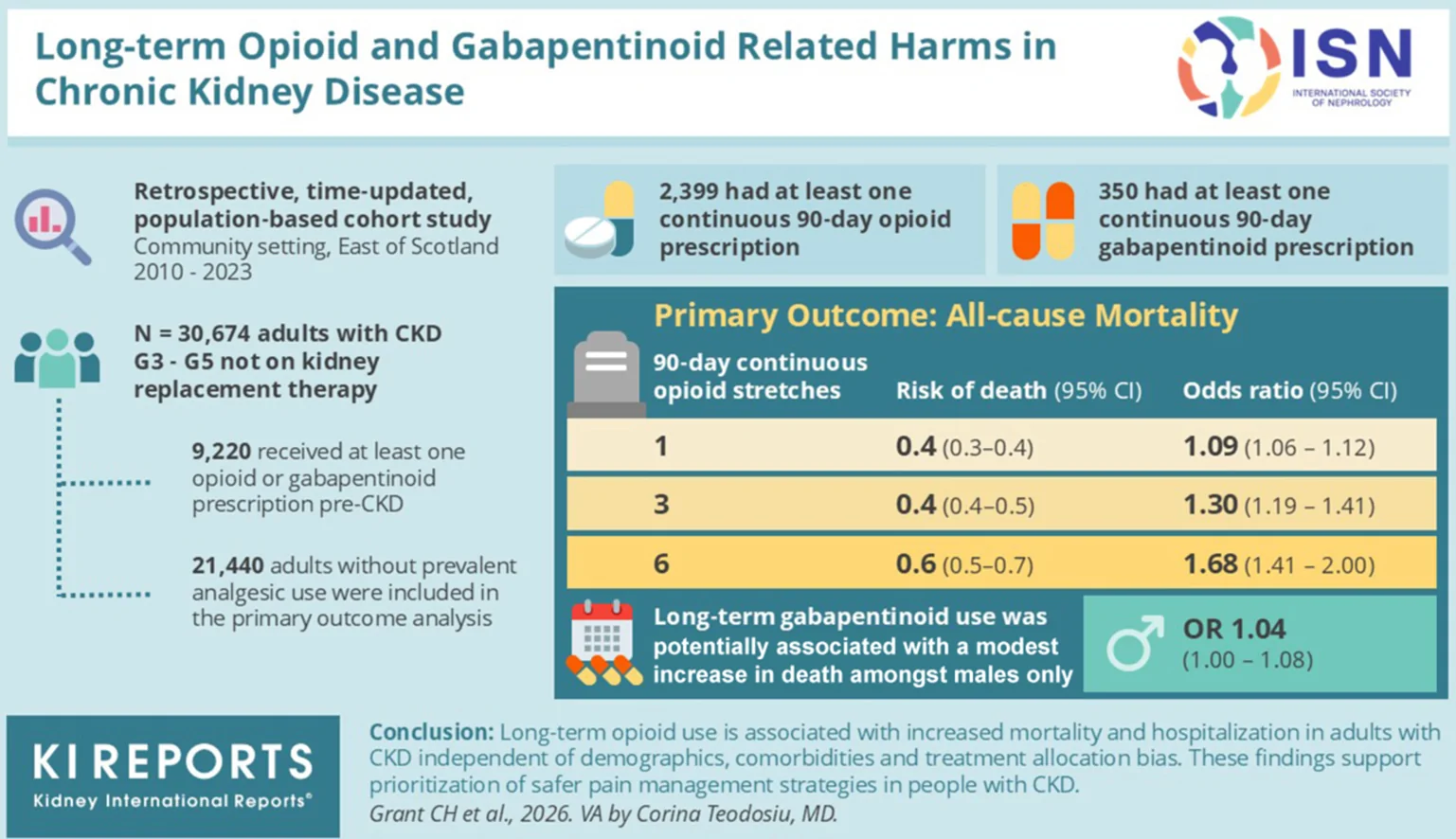

CKD affects a tenth of the global population^1^^,^^2^ and is predicted to be the fifth leading cause of death worldwide by 2040.^3^ It carries a significant symptom burden contributing to disability, reduced quality of life, and high costs to individuals and societies.^4^^,^^5^

Pain is a common and debilitating symptom complex in patients with CKD with chronic pain estimated to affect over half of the patients with CKD.^6^ Management is currently suboptimal with no international consensus guidelines to inform clinical decision making.^7^ Pharmacologic approaches are frequently based upon local expertise^8^^,^^9^ or extrapolated from general population guidance,^10^ which fail to account for altered pharmacokinetics and pharmacodynamics present with reduced kidney function. Despite a limited evidence base, a fifth of adults with CKD are prescribed chronic opioids for pain each year^8^ compared with around 1 in 20 in the general population.^11^ The prevalence of adjunctive treatments for pain in CKD, such as long-term gabapentinoids, is largely unknown.^8^^,^^12^

A recent meta-analysis of 2.3 million adults with CKD^8^ found that opioids were associated with fractures,^8^^,^^13^, ^14^, ^15^ hospitalization,^16^^,^^17^ and death.^16^, ^17^, ^18^, ^19^, ^20^, ^21^, ^22^ However, pharmacoepidemiologic studies informing these results are prone to important biases, including misclassification (i.e., measuring analgesic use at baseline, rather than over time),^23^ confounding by indication (i.e., comparing analgesic users to nonusers, rather than active controls)^24^ and prevalent user bias (i.e., including existing analgesic users at study entry).^25^ Furthermore, studies are frequently limited to acute analgesic exposure, short term follow-up and patients with advanced kidney disease. There is a lack of evidence for the safety or efficacy of gabapentinoids for chronic pain among patients with CKD despite reports of short-term neurologic sequelae.^8^ Long-term safety data relating to new opioid or gabapentinoid use in nondialysis dependent CKD, are required to prevent over- or subtherapeutic pharmacologic treatment of chronic pain, address symptom burden in CKD, and reduce iatrogenic harm.

This study aimed to determine the risk of death, hospitalization, and hip fractures associated with new long-term opioids or gabapentinoids for chronic pain in adults with CKD.

## Methods

### Study Design, Data Sources, and Setting

A retrospective, population-based cohort study was conducted using routinely collected data based on the population of Tayside and Fife, which constitutes approximately 20% of the Scottish population.^26^ Data were linked by the Health Informatics Centre using a unique national identifier (the Scottish Community Health Index)^27^ from the following sources: the Scottish Morbidity Record (SMR01), the Scottish Cancer Registry (SMR06), laboratory results, the Prescribing Information System, the Scottish Care Initiative-Diabetes Collaboration, the Scottish Renal Registry, and the National Records of Scotland Deaths Data. Details regarding data coverage and metrics are provided in the Supplementary Information S1.

### Source and Study Population and Study Period

The source population consisted of people of any age alive and a resident of Tayside or Fife on January 1, 2009 or January 1, 2014, respectively with at least 2 serum creatinine tests.^28^^,^^29^ The study period was January 1, 2010 (Tayside) or 2015 (Fife) until January 1, 2023 inclusive. The study population included adults (age ≥ 18 years) with CKD stages G3 to G5 (eGFR based on creatinine < 60 ml/min per 1.73 m^2^ based on 2009 CKD Epidemiology Collaboration creatinine equation^30^) according to the Kidney Disease: Improving Global Outcomes consensus definition^30^^,^^31^ during the study period to allow a minimum follow-up of 1 year for outcome ascertainment for all patients. Patients who died, left Tayside and/or Fife, or received kidney replacement therapy (i.e., dialysis or kidney transplantation) for kidney failure less than 1 day after CKD diagnosis were excluded. Full details are outlined in Supplementary Information S2.

### Primary Exposure Definitions and Measurement

The primary exposure of interest was new receipt and cumulative exposure to long-term (i.e., continuous 90-day prescription episodes with a permissible gap of up to 28 days) noninjectable opioids or gabapentinoids for pain. Relevant drug code lists were derived using a recommended approach (Supplementary Information S3).^32^ For each prescription, British National Formulary name, code, chapter, and date of prescription dispensation were extracted. Each prescription was assumed to be of 28 days duration from the date of dispensation. Opioids were delineated as strong or weak (Supplementary Table S1).^33^^,^^34^ Full details of primary exposure measurement are provided in Supplementary Information S4.

### Demographics and Comorbidities

Demographic characteristics included age, sex at birth, and the Scottish Index of Multiple Deprivation (SIMD) which is a multidimensional national metric of small area level deprivation.^35^ Relevant comorbidities included CKD-related conditions, pain-related conditions, and others judged to be potentially predictive of death in the context of chronic analgesic use.^36^ A list of data sources, time windows, and codes used to determine comorbidity is provided in Supplementary Table S2.^37^

### Study Entry, Follow-Up, and Outcomes

Patient follow-up was defined as follows: study entry was the date of CKD stages G3 to G5 diagnosis as identified by the date of secondary confirmatory eGFR as defined above; date of last follow-up was the earliest of the date of exit from Tayside and/or Fife health board, death, or study end (January 1, 2024). The primary outcome was all cause-mortality. Secondary outcomes were emergency hospitalization and hip fracture (Supplementary Table S3).

### Statistical Analyses

Baseline characteristics of the cohort at study entry were summarized as median (interquartile range [IQR]) for continuous variables and frequencies (percentages) for categorical variables and stratified by prevalent analgesic use. The Pearson chi-square test was used to assess between group differences for categorical variables.

#### Prevalent Analgesic Users

Six months of baseline prescribing data was used to identify prevalent opioid and/or gabapentinoid users based on any single prescription before the study entry. A 6-month period was selected to avoid any single remote analgesic prescription from excluding patients in the new user analysis, given the primary exposure of interest was long-term analgesic prescription for chronic pain.^38^^,^^39^ Prevalent opioid and/or gabapentinoid users were excluded from the primary and secondary outcome analyses to avoid prevalent user bias.^40^

#### Data Preparation for Discrete-Time Analysis

The data were prepared for discrete-time analyses and time-updated covariates using methods described previously.^41^, ^42^, ^43^ In brief, person-time was derived by separating each person’s follow-up into sequential 28-day intervals from study entry to date of the last follow-up (i.e., death, exit from health board, or study end), and their vital status was recorded for each interval. A data table was created with 1 row per 28-day interval per individual with status at the start of each interval (time t) recorded for time-fixed covariates (sex, SIMD) and time updated covariates (i.e., age, eGFR based on creatinine, years since CKD diagnosis, comorbid conditions, ever exposed to opioids or gabapentinoids by the start of the interval, and cumulative 90-day analgesic exposure). Patients therefore contributed person-time to both the analgesic exposure and nonexposure time periods across the length of their follow-up from entry to last follow-up.^41^^,^^44^ For each drug class, the exposure duration was deduced from the first and last dates of a run of repeat prescriptions for a relevant analgesic plus the duration of the last prescription in that run. Cumulative 90-day exposure spells were summed and time-updated (i.e., cumulative exposure at time t).

#### Primary Analysis: Discrete Time Logistic Model

A discrete time logistic model was used to estimate the odds of dying within each 28-day interval and assess the impact of cumulative analgesic exposure on mortality risk among new analgesic users. These methods have been employed previously^41^, ^42^, ^43^^,^^45^ and are described in detail in Supplementary Information S5.

In brief, ever and never analgesic users differ in terms of measured and unmeasured confounders related to frailty. To allow for this treatment allocation bias, 2 time-updated terms for analgesic exposure were included in the model, as follows: an ever-exposure and a cumulative exposure term.^42^ The former captures time-invariant treatment allocation bias and any immediate effect of the drug, should it exist, in which case interpretation of drug harms based on the cumulative (i.e., 90-day) exposure term would be conservative. To determine analgesic related harms for chronic pain management, rather than sporadic use, analgesic exposure was quantified in terms of the number of the previous continuous 90-day spells.

To inform the final analyses, direct acyclic graphs were created (Supplementary Information S6, Supplementary Figures S1 and S2).^46^^,^^47^ Minimally adjusted models (current age and baseline sex) were created. The final model included time dependent terms for duration of CKD diagnosis, age, an interaction term (age × CKD duration), eGFR based on creatinine, cardiovascular disease, cancer, depression, respiratory disease, diabetes, ever previous analgesic use, baseline sex, and socioeconomic status. Models were also compared when stratified by sex.

Generalized estimating equations logistic regression was used to account for correlation between repeated measures (recorded every 28 days) within individuals.^48^ A nonlinear association between cumulative analgesic use and adverse outcomes was considered clinically plausible hence analgesic exposure terms were modelled using linear and quadratic terms with the quasi-likelihood under the independence model criterion compared among each exposure-outcome pair.^49^ Where quadratic terms were selected because of improved model fit, marginal standardization was used to present population-average absolute and relative risks across exposure levels.

Secondary outcomes were analyzed using the same approach; however, Poisson regression was applied to emergency hospitalization. Patients with missing SIMD were excluded from the primary analysis, because there were very few missing cases (< 0.1%) and missingness likely reflected administrative rather than patient related factors.

Multiple sensitivity analyses were performed including varying the applied definitions of CKD diagnosis, prevalent analgesic use, prescription duration, and permissible gap in addition to a negative control exposure analysis to test whether the results were because of unmeasured confounding (Supplementary Information S7).

All analyses were conducted using R statistical software programming (version 4.4.1; R Studio, Boston, MA) using the geepack package (version 1.3.13).^50^

### Ethical Approval and Reporting

The study was approved by the East of Scotland Research Ethics Committee (REC Ref: 22/ES/0034), prospectively preregistered in the Open Science Framework (osf.io/5qsyh) and reported with reference to the Strengthening the Reporting of Observational studies in Epidemiology^51^ and Reporting of studies Conducted using Observational Routinely-collected health Data for Pharmacoepidemiology checklists.^52^

### Patient and Public Involvement

The study was supported by a patient and public involvement group, of which each member was renumerated in line with National Institute for Health and Care Research guidance. At the feasibility stage, before data application, the patient and public involvement group reviewed and discussed the importance of the research objectives. At the end of the study, feedback was offered on how the results might affect patients. Our patient and public involvement and engagement group edited our lay summary and will contribute to our dissemination plans, including social media posts and infographics upon publication.

## Results

### Study Population, Prevalent Analgesic Use, and Overall Mortality

The study population comprised 30,674 adults with CKD diagnosed during the study period (Figure 1).

**Figure 1:**
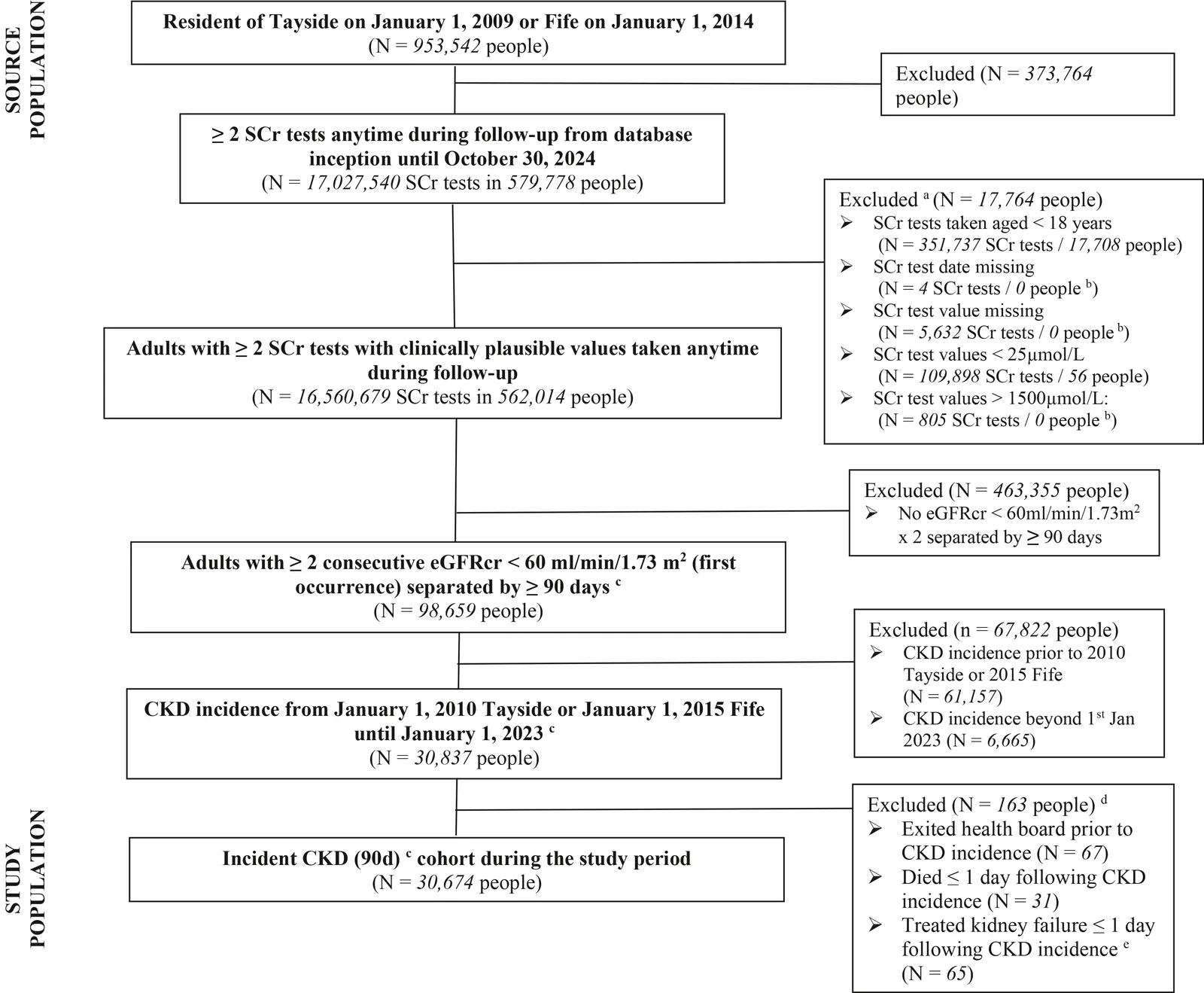
Study flow diagram outlining reasons for inclusion/exclusion from source to study population. ^a^Reasons for excluding SCR tests were not mutually exclusive. ^b^No people were excluded as all had other SCr tests with values specified within the range of clinical plausibility. ^c^The date of the second confirmatory eGFRcr (CKD-EPI 2009 without race) was the date of “CKD incidence,” that is, entry into the study population. ^d^Reasons for excluding were made sequentially in the order specified. ^e^Date of treated kidney failure refers to the first date HD/PD/Tx for end staged kidney disease based on the SRR. CKD, chronic kidney disease; CKD-EPI, Chronic Kidney Disease Epidemiology Collaboration; eGFRcr, estimated glomerular filtration based on creatinine; HD, hemodialysis; PD, peritoneal dialysis; SCr, serum creatinine; SRR, Scottish Renal Registry; Tx, transplantation.

In the 6 months before CKD diagnosis, 8508 (27.7%) and 1949 (6.4%) of 30,674 had 1 or more opioid or gabapentinoid prescription, respectively. Overall, 9220 (30.1%) patients received at least 1 of these analgesic classes (i.e., prevalent analgesic users). Table 1 describes the baseline demographic and clinical characteristics of the study cohort, stratified by prevalent analgesic use at baseline. Opioid and gabapentinoid subtypes prescribed in the 6 months before study entry are shown in Supplementary Tables S4 and S5. The most frequently prescribed opioid items were cocodamol, tramadol, and codeine, whereas gabapentin was prescribed more often than pregabalin. Among 8508 opioid users at baseline, the median number of opioid prescriptions was 3 (IQR: 5; range: 1–39) and 1277 (15.0% of opioid users) people received at least 1 strong opioid prescription. Among 1949 gabapentinoid users at baseline, the median number of gabapentinoid prescriptions was 5 (IQR: 3; range: 1–22). SIMD status was missing in 22 people, but otherwise, no missing data were identified.

**Table 1:** Baseline demographic and clinical characteristics of the study cohort stratified by baseline analgesic use

Category	Study population (*N* = 30,674)	No opioids or gabapentinoids (*n* = 21,454)	Opioids or gabapentinoids (*n* = 9220)	*P*-value
Sex				
F	15,781 (51.4)	10,382 (48.4)	5399 (58.6)	< 0.001
M	14,893 (48.6)	11,072 (51.6)	3821 (41.4)	
Age (yrs)				
Median (IQR)	75.2 (68.3–81.6)	75.7 (69.0–82.1)	74.1 (66.8–80.5)	< 0.001
Age category				
<45	412 (1.3)	310 (1.4)	102 (1.1)	< 0.001
45-54	1041 (3.4)	634 (3.0)	407 (4.4)	
55-64	3718 (12.1)	2310 (10.8)	1408 (15.3)	
65-74	9845 (32.1)	6797 (31.7)	3048 (33.1)	
75-84	11,220 (36.6)	8062 (37.6)	3158 (34.3)	
85+	4438 (14.5)	3341 (15.6)	1097 (11.9)	
Deprivation (SIMD quintile)				
1	5219 (17.0)	3232 (15.1)	1987 (21.6)	< 0.001
2	5804 (18.9)	3774 (17.6)	2030 (22.0)	
3	6413 (20.9)	4576 (21.3)	1837 (19.9)	
4	8223 (26.8)	6055 (28.2)	2168 (23.5)	
5	4993 (16.3)	3803 (17.7)	1190 (12.9)	
Missing	22 (0.1)	14 (0.1)	8 (0.1)	
Health board				
F	11,752 (38.3)	8171 (38.1)	3581 (38.8)	0.218
T	18,922 (61.7)	13,283 (61.9)	5639 (61.2)	
eGFR at diagnosis				
Median (IQR)	53.4 (47.8–57.0)	53.6 (48.2–57.0)	52.8 (46.8–56.7)	< 0.001
eGFR category				
G3a	25233 (82.3)	17884 (83.4)	7349 (79.7)	< 0.001
G3b	4365 (14.2)	2883 (13.4)	1482 (16.1)	
G4	854 (2.8)	548 (2.6)	306 (3.3)	
G5	222 (0.7)	139 (0.6)	83 (0.9)	
Cardiovascular disease	10,970 (35.8)	7200 (33.6)	3770 (40.9)	< 0.001
Diabetes	6338 (20.7)	4055 (18.9)	2283 (24.8)	< 0.001
Cancer	3726 (12.1)	2327 (10.8)	1399 (15.2)	< 0.001
Depression	7881 (25.7)	4078 (19.0)	3803 (41.2)	< 0.001
Chronic liver disease	450 (1.5)	270 (1.3)	180 (2.0)	< 0.001
Chronic respiratory disease	6543 (21.3)	3841 (17.9)	2702 (29.3)	< 0.001
Rheumatoid arthritis	297 (1.0)	125 (0.6)	172 (1.9)	< 0.001
Opioid use disorder	48 (0.2)	21 (0.1)	27 (0.3)	< 0.001

eGFR, estimated glomerular filtration rate; IQR, interquartile range; SIMD, Scottish Index of Multiple Deprivation.

### Cumulative Analgesic Exposure and Length of Follow-Up

A total of 21,440 patients without prevalent analgesic use and with nonmissing SIMD were included in the primary and secondary analyses and followed up for a median of 4.3 years (IQR: 2–7). Analgesic exposure during follow-up is summarized in Supplementary Table S6.

### Analgesic Use and All-Cause Mortality

Among 21,440 patients included in the primary outcome analysis, 7956 died, 179 left the health board area, and 13,305 were alive at study end. Among 2399 patients with at least 1 continuous 90-day stretch of opioids during follow-up, 1159 patients died compared with 10 who left the health board and 1230 who were alive at the end of the study. Those who died had a median of 88 days (IQR: 43–430) from date of last completed 90-day opioid stretch until death. In a discrete time logistic regression model (*n* = 21,440), the relationship between cumulative 90-day opioid use and death was nonlinear; hence, the quadratic exposure term was applied with marginal standardization used to present absolute and relative risks (Table 2, Figure 2 and Supplementary Table S7). The adjusted OR (aOR) for death increased as the number of continuous 90-day opioid stretches increased from 1 to 6. The absolute risk of death was higher and relative impact of cumulative opioid exposure more pronounced among men than in women (Supplementary Table S8 and Supplementary Figures S3 and S4).

**Table 2:** Marginal risk of death and odd ratios by long-term 90-day cumulative opioid exposure

90-d continuous opioid stretches	Risk of death (95% CI)	OR (95% CI)	*P*-value
0	0.3 (0.3–0.3)	1.00 (1.00–1.00)	NA
1	0.4 (0.3–0.4)	1.09 (1.06–1.12)	< 0.001
2	0.4 (0.4–0.4)	1.19 (1.12–1.26)	< 0.001
3	0.4 (0.4–0.5)	1.30 (1.19–1.41)	< 0.001
4	0.5 (0.4–0.5)	1.41 (1.26–1.59)	< 0.001
5	0.5 (0.4–0.6)	1.54 (1.33–1.78)	< 0.001
6	0.6 (0.5–0.7)	1.68 (1.41–2.00)	< 0.001

CI, confidence interval; CKD, chronic kidney disease; eGFR_cr_, estimated glomerular filtration rate based on creatinine; GEE, generalized estimating equations; OR, odds ratio; SMID, Scottish Index of Multiple Deprivation.

Based on *n* = 21,440 nonprevalent opioid or gabapentinoid users at baseline without missing SIMD. Probability of death during the 28-day time interval having survived to the start of that interval derived from a GEE logistic model including the following terms: cumulative 90-day opioid use (linear), cumulative 90-day opioid use (quadratic), duration of CKD diagnosis (years, centered, linear), current age (years, centered, linear), age x CKD duration interaction, eGFRcr (linear), cardiovascular disease, cancer, depression, respiratory disease, diabetes, ever previous opioid use, ever previous gabapentinoid use, baseline sex (fixed), and socioeconomic status (fixed). OR are relative to an exposure of 0. Covariates time-updated unless otherwise specified. *P*-values are Wald tests comparing each opioid exposure level with the reference level derived from the fitted GEE model.

**Figure 2:**
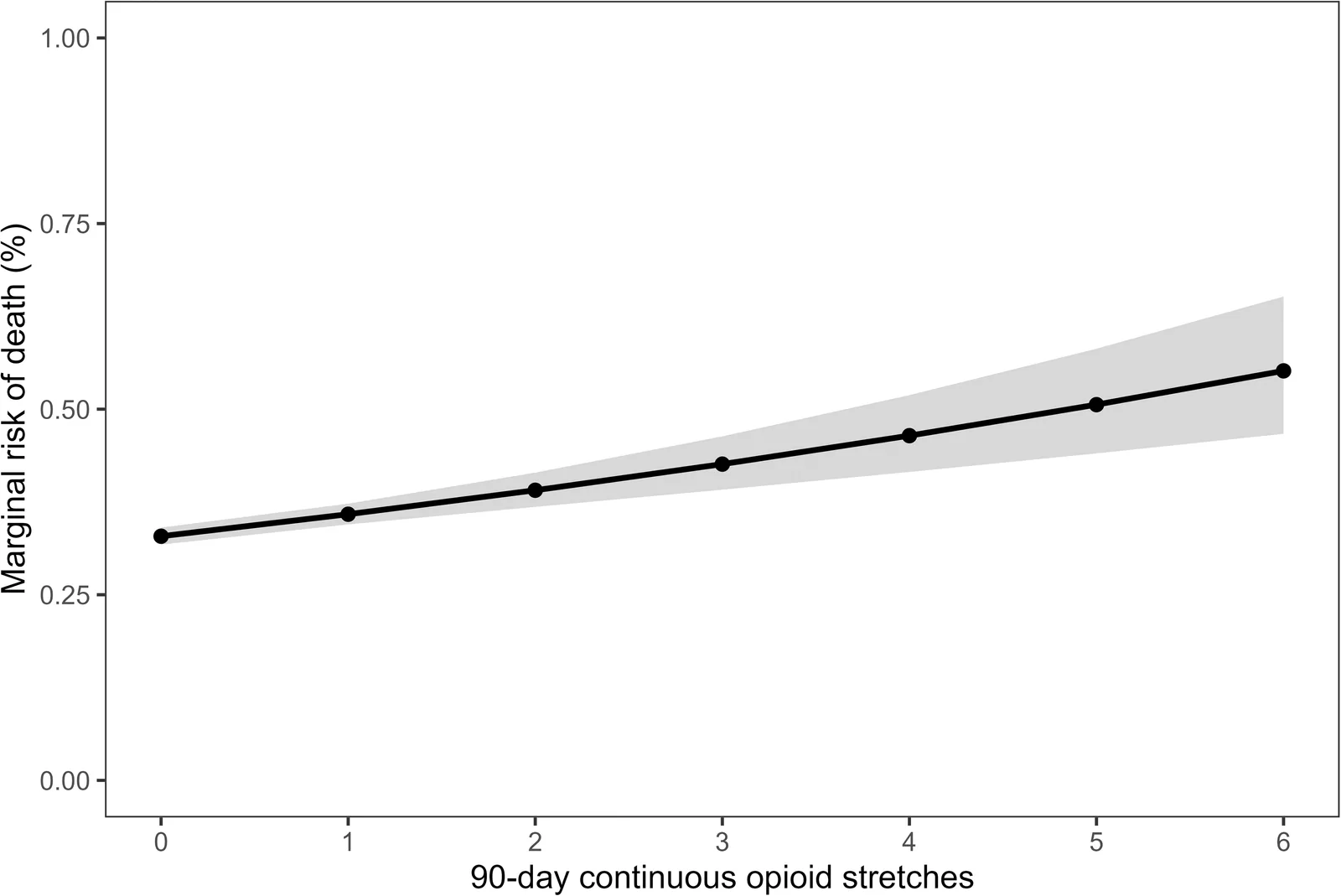
Marginal risk of death by long-term 90-day cumulative opioid exposure.

Among 350 patients with at least 1 continuous 90-day stretch of gabapentinoids during follow-up, 140 patients died compared with 210 who were either alive at study end or who left health board (<5). Those who died had a median of 190 days (IQR: 62–964) from date of last completed 90-day gabapentinoid stretch until death. Cumulative gabapentinoid exposure was associated with mortality with each additional 90-days of cumulative use being associated with a modestly increased odds of death (Table 3, Figure 3, and Supplementary Table S9). This association was apparent among males, but not females (Supplementary Table S10).

**Table 3:** Multivariable adjusted association between long-term 90-day cumulative gabapentinoid exposure and all-cause mortality

Variable	OR (95% CI)	*P*-value
Age (current, yrs)	1.09 (1.08–1.09)	< 0.001
Female sex	0.85 (0.81–0.90)	< 0.001
CKD duration (yrs)	0.90 (0.89–0.91)	< 0.001
Age x CKD duration interaction	1.00 (1.00–1.00)	< 0.001
SIMD 3 (baseline)	0.92 (0.86–0.99)	0.019
SIMD 4-5 (baseline)	0.86 (0.81–0.91)	< 0.001
Cardiovascular disease	2.07 (1.96–2.17)	< 0.001
Cancer	2.34 (2.21–2.47)	< 0.001
Depression	1.52 (1.44–1.61)	< 0.001
Diabetes mellitus	1.20 (1.13–1.27)	< 0.001
Chronic respiratory disease	1.37 (1.30–1.45)	< 0.001
eGFR creatinine	0.99 (0.99–0.99)	< 0.001
Gabapentinoid ever use	0.91 (0.80–1.03)	0.150
Opioid ever use	1.61 (1.53–1.70)	< 0.001
Cumulative 90 d gabapentinoid use	1.03 (1.00–1.05)	0.038

CI, confidence interval; CKD, chronic kidney disease; eGFR, estimated glomerular filtration rate; OR, odds ratio; SMID, Scottish Index of Multiple Deprivation.

Based on *n* = 21,440 nonprevalent opioid or gabapentinoid users at baseline without missing SIMD. GEE logistic model including the following terms: cumulative 90-day gabapentinoid use (linear), duration of CKD diagnosis (years, centered, linear), current age (years, centered, linear), age x CKD duration interaction, eGFRcr (linear), cardiovascular disease, cancer, depression, respiratory disease, diabetes, ever previous opioid use, ever previous gabapentinoid use, baseline sex (fixed), and socioeconomic status (fixed). Estimates shown are ORs with Wald 95% CIs and *P*-values. Each variable is time-updated unless otherwise specified.

**Figure 3:**
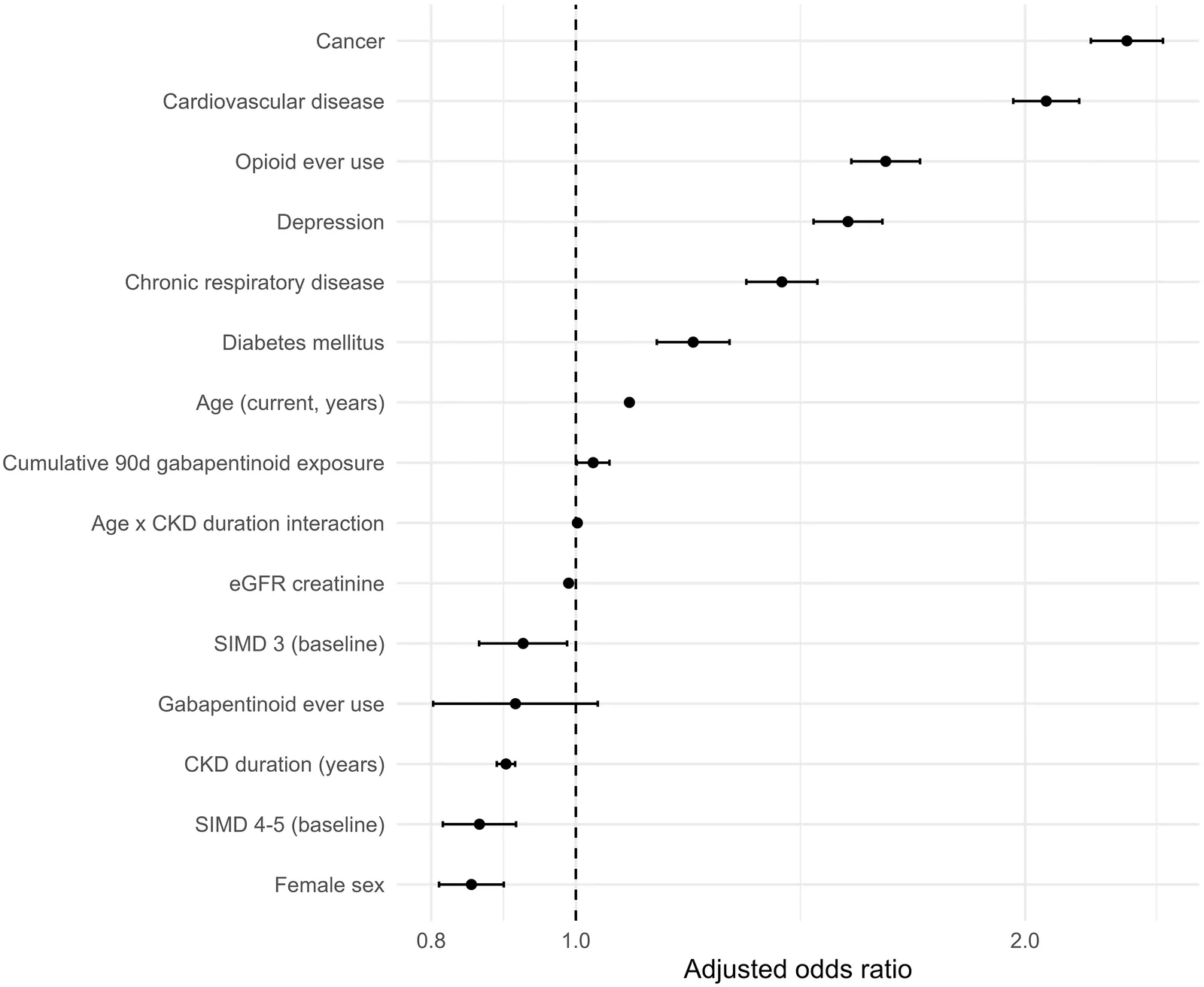
Forest plot of the association between long-term 90-day cumulative gabapentinoid exposure and all-cause mortality. CKD, chronic kidney disease; eGFR, estimated glomerular filtration rate; SIMD, Scottish Index of Multiple Deprivation.

### Analgesic Use, Emergency Hospitalizations, and Hip Fractures

There were 75,018 emergency hospitalizations among 12,896 patients (60.1% [12,896/21,440]) during follow-up. Among 2399 people with at least 90-days of cumulative opioid exposure, 1897 (79.1% [1,897/2,399]) had at least 1 emergency hospitalization compared with 10,999 of 19,041 (57.8%) of those without. There was a modest graded relationship between cumulative opioid exposure and emergency hospitalization (Table 4 and Figure 4). The absolute risk of hospitalization and relative impact of cumulative opioid exposure appeared more pronounced amongst men and only significant amongst women after four or more completed opioid stretches (Supplementary Table S11 and Supplementary Figures S5 and S6). Among 350 patients with at least 1 stretch of 90-days of gabapentinoid use, 275 (78.6% [275/350]) had 1 or more hospital admission compared with 12,621 of 21,090 (59.8%) of those without gabapentanoid use. Cumulative gabapentinoid exposure was associated with a modest increase in emergency hospitalization in men, but not women (Supplementary Table S12).

**Table 4:** Marginal rate of emergency hospitalization and rate ratios by 90-day cumulative opioid exposure

90-d continuous opioid stretches	Emergency hospitalization rate (per 100 person-years)	Rate ratio (95% CI)	*P*-value
0	32.86 (32.44–33.30)	1.00 (1.00–1.00)	NA
1	34.07 (33.58–34.56)	1.04 (1.03–1.05)	< 0.001
2	35.31 (34.56–36.08)	1.07 (1.05–1.10)	< 0.001
3	36.61 (35.51–37.74)	1.11 (1.08–1.15)	< 0.001
4	37.95 (36.45–39.50)	1.15 (1.11–1.20)	< 0.001
5	39.34 (37.41–41.36)	1.20 (1.14–1.26)	< 0.001
6	40.78 (38.39–43.31)	1.24 (1.17–1.32)	< 0.001

CI, confidence interval; CKD, chronic kidney disease; eGFR_cr_, estimated glomerular filtration rate based on creatinine; GEE, generalized estimating equations; RR, rate ratio; SMID, Scottish Index of Multiple Deprivation.

Based on *n* = 21,440 nonprevalent opioid or gabapentinoid users at baseline without missing SIMD. Event rate during the 28-day time interval having survived to the start of that interval derived from a GEE Poisson model including the following terms: cumulative 90-day opioid use (linear), cumulative 90-day opioid use (quadratic), duration of CKD diagnosis (years, centered, linear), current age (years, centered, linear), age x CKD duration interaction, eGFRcr (linear), cardiovascular disease, cancer, depression, respiratory disease, diabetes, ever previous opioid use, ever previous gabapentinoid use, baseline sex (fixed), and socioeconomic status (fixed). RR are relative to an exposure of 0. Covariates time-updated unless otherwise specified. *P*-values are Wald tests of the rate-ratio contrast comparing each opioid exposure level with the reference level derived from the fitted GEE model.

**Figure 4:**
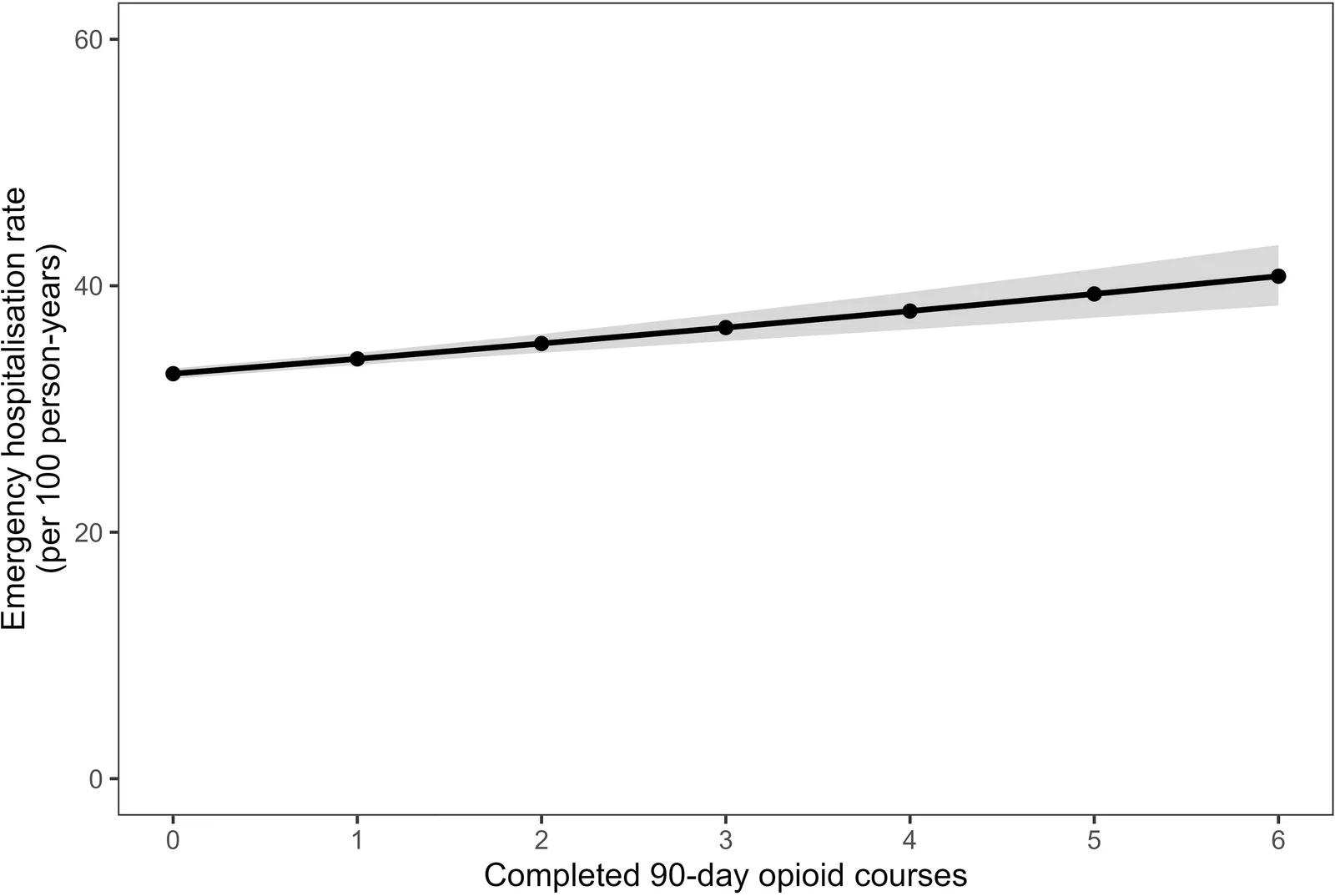
Marginal risk of emergency hospitalization by long-term 90-day cumulative opioid exposure.

There were 783 hip fracture events in 728 people during follow-up. Among 2399 people with at least 1 stretch of 90-days of opioid use, 151 had 1 or more hip fractures (6.3%) compared with 577 of 19,041 (3.0%) without 90-days of cumulative opioid use. Nine of 350 patients (2.6%) with at least 1 stretch of 90-days of gabapentinoids had a hip fracture compared with 719 of 21,090 (3.4%) of those without. There was no association observed between cumulative opioids or gabapentinoids and hip fractures (Supplementary Tables S13 and S14).

### Sensitivity Analyses

There were 46,459 diagnosed with renal dysfunction who entered the study among whom 15,744 had prevalent analgesic use at baseline. There was a stronger association between cumulative opioid exposure and death than in the primary study cohort (aOR: 1.11 [1.08–1.14] to 1.87 [1.62–2.16] for 1 to 6 continuous 90-day opioid stretches). There was no relationship between cumulative gabapentinoid use and mortality (aOR: 1.026 [0.998–1.054]). Exclusion of established prevalent users at baseline (n = 4693) led to an attenuated relationship between opioids and death (aOR: 1.06 [1.04–1.07] to 1.39 [1.25–-1.54] for 1 to 6 continuous 90-day opioid stretches) without attenuation in the association between gabapentinoids and mortality (aOR: 1.019 [1.000–1.039]). The association between opioids and mortality persisted when both the assumed prescription duration was increased (aOR: 1.05 [1.03–1.07] to 1.35 [1.20–1.52]), and the permissible gap reduced (aOR: 1.12 [1.08–1.17] to 2.02 [1.59–2.56]) for 1 to 6 continuous 90-day opioid stretches, respectively. Gabapentinoids were not associated with increased mortality when assumed prescription duration (1.016 [0.995–1.037]) or permissible gap were altered (1.027 [0.997–1.058]). In a negative control exposure analysis, 90-day continuous stretches of lipid-lowering therapy were associated with a lower odds of death and a static absolute marginal risk of death (Supplementary Table S15).

## Discussion

In a population-based study of adults with CKD, new long-term cumulative opioid use was associated with increased emergency hospitalization and death. These results were unexplained by analgesic indication, demographic and/or clinical characteristics, or unmeasured confounding. The relationship between chronic opioids, hospitalization, and mortality was evident in both sexes in a dose-dependent manner, but appeared more pronounced among men who had a higher baseline risk of adverse outcomes. Chronic gabapentinoid exposure was not consistently associated with increased mortality in both sexes or across all sensitivity analyses, despite evidence of increased harms (i.e., emergency hospitalization and death) among men. Neither opioids, nor gabapentinoids, were independently associated with hip fractures.

In a meta-analysis^8^ we previously reported that opioid use was associated with increased mortality (n = 7 studies),^16^, ^17^, ^18^, ^19^, ^20^, ^21^, ^22^ however, only 3 studies measured chronic (≥ 90-days) exposure.^16^^,^^20^^,^^22^ Abbott^20^ and Kimmel *et al.*^16^ reported chronic opioids to be associated with increased deaths in kidney transplant and dialysis recipients with effect sizes similar to our findings.^16^^,^^20^ Han *et al.*^36^ reported a higher risk of death among ‘ever opioid users’ compared with nonusers among older adults with CKD; however, the results were confounded by indication. Zhan *et al.*^21^ prospectively followed up patients with nondialysis CKD and found that opioids were associated with increased death compared with nonsteroidal anti-inflammatory drugs. However, opioid use was assessed once a year based on the preceding 30-days; hence, the results were subject to misclassification bias.^21^ Despite the limitations outlined, previously published results are broadly comparable with our findings.

Our meta-analysis^8^ also identified an association between high dose opioid use and hospitalization (summary hazards ratio: 1.38), based on 2 studies.^16^^,^^17^ By comparison, our effect sizes were modest, which may reflect that our exposure included any opioid dose. Previous studies of older adults with CKD have reported high dose gabapentinoids to be associated with emergency hospitalization,^53^ encephalopathy^53^^,^^54^ and falls,^53^ but not fracture or respiratory depression.^53^ However, outcomes were short-term (i.e., 30-days), and the results were not generalizable to nonelderly patients.^53^^,^^54^

Our study has notable strengths including the use of population-based data with prolonged follow-up. We used a biochemical definition of CKD, consistent with consensus guidelines and used nationally representative data sets with high population coverage. We took steps to minimize bias through time-updated exposure assessment, exclusion of baseline prevalent users, and use of the ever-analgesic term to address treatment allocation bias. Our use of a negative control outcome analysis did not suggest that the primary findings were related to unmeasured confounding. Finally, we evaluated nonkidney replacement treatment dependent CKD which represents the majority of CKD at a population level.

Our study has important limitations. We could not assess the benefits of analgesics. Missing analgesic dose, directions, and indication(s) limited the evaluation of granular exposure estimates. Furthermore, there is a potential for exposure misclassification because of the need to assume a prescription duration of 28 days. Specific opioid subtypes could not be evaluated because of an insufficient sample size to correctly model a multitude of potential analgesic exposures over cumulative 28-day intervals. Prescription dispensation does not necessarily reflect actual medication use, although it is a widely accepted proxy for consumption.^11^ There remains a potential for time-dependent treatment allocation bias because of unmeasured time-varying confounders, because long-term analgesics are more likely to be prescribed when patients develop greater symptom burden, multimorbidity, and frailty over time. Approaches such as marginal structural modelling can account for time varying confounders affected by previous treatment. However, standard marginal structural modelling assumes no unmeasured confounders, whereas unmeasured confounding is expected in routine healthcare data. Our approach allows for treatment allocation bias because of both measured and unmeasured time-invariant confounders associated with starting analgesics, has the advantage of relative simplicity, and is more likely to underestimate analgesic harms because of overadjustment, making our conclusions conservative. Albuminuria data was unavailable to identify CKD potentially leading to misclassification. Finally, we did not have access to ethnicity data; however, our results reflect a Scottish population of predominantly White ethnicity and may not be generalizable to multiethnic groups.

The findings provide compelling evidence of harms related to long-term opioid use. For example, an individual patient with CKD prescribed chronic opioids for 18 months may experience a 2-fold relative increase in mortality. The results are important for primary care practitioners, who manage most patients with nonkidney failure CKD, and suggest the need to consider active deprescribing of long-term opioids after considering an individual patient’s risk-benefit ratio. This approach is consistent with guidance in the general population which increasingly favors nonpharmacologic, multimodal approaches and recommend against long term opioid prescribing for chronic pain.^55^^,^^56^ When pharmacologic treatments are warranted, frequent and early clinical review is required.^56^

These results are relevant for policymakers when considering the availability of multidisciplinary chronic pain specialist services and provision of nonpharmacologic treatments including physical therapy,^57^ psychological treatments,^58^ and self-management techniques.^56^^,^^59^, ^60^, ^61^ Individualized approaches are required to address a biopsychosocial model of chronic pain, including realistic goal setting in partnership with patients to promote self-efficacy and increase life participation.^56^

Future work should focus on the risk-benefit ratio of pharmacologic treatments for chronic pain in adults with CKD, particularly in relation to patient reported outcome measures. In addition, future studies should corroborate and extend our findings to compare opioid subtypes and other adjunctive therapies and assess cause-specific hospitalization and/or mortality. There is a dearth of evidence to support nonpharmacologic interventions for chronic pain in CKD which must be addressed given the rising prevalence of CKD and potential for analgesic-related harms.

In conclusion, long-term opioids are associated with increased emergency hospitalizations and mortality in adults with CKD. Gabapentinoids are potentially associated with similar harms amongst men. Further research is required to establish optimal approaches to manage chronic pain in adults with CKD.

## Disclosure

SB has received a grant from AstraZeneca and consultancy fees from AstraZeneca, GSK, Stada UK, Novo Nordisk, and Bayer. PBM has received grants from AstraZeneca and Boehringer Ingelheim; consulting fees from AstraZeneca, Boehringer Ingelheim, Pharmacosmos, Vifor, and Stada UK; payment or honoraria from AstraZeneca, Boehringer Ingelheim, Pharmacosmos, Bayer, and Vifor and is a member of the data safety monitoring or advisory board of Vertex and Novartis. LAC receives research support funding, on behalf of her institution, from the Advanced Pain Discovery Platform (funded by UK Research and Innovation, Versus Arthritis, and Eli Lilly), the Scottish Government (Chief Scientist Office), the Wellcome Trust, and the National Institute of Academic Anaesthesia. LAC is also the Vice Chair of the Scottish Intercollegiate Guidelines Network Council and chair of the Scottish Intercollegiate Guidelines Network Guideline Development Group for Management of Chronic Pain.

The other authors declared no competing interests.

## Supplementary Material

Supplementary file (PDF) **Supplementary Reference. RECORD-PE statement. Supplementary Information S1.** Data sources and access. **Supplementary Information S2.** Inclusion and exclusion criteria. **Supplementary Information S3.** Code list generation and clinician review. **Supplementary Information S4.** Prescription duration and permissible gap. **Supplementary Information S5.** Discrete time logistic model. **Supplementary Information S6.** Direct acyclic graph creation. **Supplementary Information S7.** Sensitivity analyses. **Figure S1.** Direct acyclic graph depicting the proposed relationship between chronic opioid exposure and mortality. **Figure S2.** Direct acyclic graph depicting the proposed relationship between chronic gabapentinoid exposure and mortality. **Figure S3.** Marginal risk of death by cumulative opioid exposure in females. **Figure S4.** Marginal risk of death by cumulative opioid exposure in males. **Figure S5.** Marginal rate of emergency hospitalization by cumulative opioid exposure in females. **Figure S6.** Marginal rate of emergency hospitalization by cumulative opioid exposure in males. **Table S1.** List of relevant opioids and gabapentinoids. **Table S2.** List of data sources, time windows and relevant codes to determine co-morbidity at baseline. **Table S3.** List of data sources, relevant codes and supporting references to determine cause-specific secondary outcomes. **Table S4.** Opioid subtypes among patients with baseline 6 months opioid use. **Table S5.** Gabapentinoid subtypes among patients with baseline 6 months gabapentinoid use. **Table S6.** Opioid and gabapentinoid exposure during follow-up. **Table S7.** Marginal risk of death and odd ratios by 90-day cumulative opioid exposure (minimally adjusted). **Table S8.** Marginal risk of death and odd ratios by 90-day cumulative opioid exposure stratified by sex. **Table S9.** Association between long-term 90-day cumulative gabapentinoid exposure and all-cause mortality (minimally adjusted). **Table S10.** Association between long-term 90-day cumulative gabapentinoid exposure and all-cause mortality stratified by sex. **Table S11.** Marginal rate of emergency hospitalization and adjusted rate ratios by 90-day cumulative opioid exposure stratified by sex. **Table S12.** Association between long-term 90-day cumulative gabapentinoid exposure and emergency hospitalization stratified by sex. **Table S13.** Association between long-term 90-day cumulative opioid exposure and hip fractures. **Table S14.** Association between long-term 90-day cumulative gabapentinoid exposure and hip fractures. **Table S15.** Marginal risk of death and odd ratios by 90-day cumulative lipid-lowering therapy.

## Footnotes

Supplementary file (PDF)

**Supplementary Reference.**

**RECORD-PE statement.**
**Supplementary Information S1.** Data sources and access. **Supplementary Information S2.** Inclusion and exclusion criteria. **Supplementary Information S3.** Code list generation and clinician review. **Supplementary Information S4.** Prescription duration and permissible gap. **Supplementary Information S5.** Discrete time logistic model. **Supplementary Information S6.** Direct acyclic graph creation. **Supplementary Information S7.** Sensitivity analyses. **Figure S1.** Direct acyclic graph depicting the proposed relationship between chronic opioid exposure and mortality. **Figure S2.** Direct acyclic graph depicting the proposed relationship between chronic gabapentinoid exposure and mortality. **Figure S3.** Marginal risk of death by cumulative opioid exposure in females. **Figure S4.** Marginal risk of death by cumulative opioid exposure in males. **Figure S5.** Marginal rate of emergency hospitalization by cumulative opioid exposure in females. **Figure S6.** Marginal rate of emergency hospitalization by cumulative opioid exposure in males. **Table S1.** List of relevant opioids and gabapentinoids. **Table S2.** List of data sources, time windows and relevant codes to determine comorbidity at baseline. **Table S3.** List of data sources, relevant codes and supporting references to determine cause-specific secondary outcomes. **Table S4.** Opioid subtypes among patients with baseline 6 months opioid use. **Table S5.** Gabapentinoid subtypes among patients with baseline 6 months gabapentinoid use. **Table S6.** Opioid and gabapentinoid exposure during follow-up. **Table S7.** Marginal risk of death and odd ratios by 90-day cumulative opioid exposure (minimally adjusted). **Table S8.** Marginal risk of death and odd ratios by 90-day cumulative opioid exposure stratified by sex. **Table S9.** Association between long-term 90-day cumulative gabapentinoid exposure and all-cause mortality (minimally adjusted). **Table S10.** Association between long-term 90-day cumulative gabapentinoid exposure and all-cause mortality stratified by sex. **Table S11.** Marginal rate of emergency hospitalization and adjusted rate ratios by 90-day cumulative opioid exposure stratified by sex. **Table S12.** Association between long-term 90-day cumulative gabapentinoid exposure and emergency hospitalization stratified by sex. **Table S13.** Association between long-term 90-day cumulative opioid exposure and hip fractures. **Table S14.** Association between long-term 90-day cumulative gabapentinoid exposure and hip fractures. **Table S15.** Marginal risk of death and odd ratios by 90-day cumulative lipid-lowering therapy.

## References

[1] Mark P.B., Stafford L.K., Grams M.E. (2025). Global, regional, and national burden of chronic kidney disease in adults, 1990-2023, and its attributable risk factors: a systematic analysis for the Global Burden of Disease Study 2023. Lancet.

[2] Duff R., Awofala O., Arshad M.T. (2024). Global health inequalities of chronic kidney disease: a meta-analysis. Nephrol Dial Transplant.

[3] Foreman K.J., Marquez N., Dolgert A. (2018). Forecasting life expectancy, years of life lost, and all-cause and cause-specific mortality for 250 causes of death: reference and alternative scenarios for 2016–40 for 195 countries and territories. Lancet.

[4] Francis A., Harhay M.N., Ong A.C.M. (2024). Chronic kidney disease and the global public health agenda: an international consensus. Nat Rev Nephrol.

[5] Torrance N., Veluchamy A., Zhou Y. (2020). Trends in gabapentinoid prescribing, co-prescribing of opioids and benzodiazepines, and associated deaths in Scotland. Br J Anaesth.

[6] Lambourg E., Colvin L., Guthrie G. (2021). The prevalence of pain among patients with chronic kidney disease using systematic review and meta-analysis. Kidney Int.

[7] Davison S.N. (2005). Chronic pain in end-stage renal disease. Adv Chronic Kidney Dis.

[8] Lambourg E., Colvin L., Guthrie G., Walker H., Bell S. (2022). Analgesic use and associated adverse events in patients with chronic kidney disease: a systematic review and meta-analysis. Br J Anaesth.

[9] Murtagh F.E., Chai M.O., Donohoe P., Edmonds P.M., Higginson I.J. (2007). The use of opioid analgesia in end-stage renal disease patients managed without dialysis: recommendations for practice. J Pain Palliat Care Pharmacother.

[10] Ventafridda V., Saita L., Ripamonti C., De Conno F. (1985). WHO guidelines for the use of analgesics in cancer pain. Int J Tissue React.

[11] Macfarlane G.J., Beasley M., Jones G.T., Stannard C. (2020). The epidemiology of regular opioid use and its association with mortality: prospective cohort study of 466 486 UK Biobank participants. EClinicalmedicine.

[12] Davison S.N., Rathwell S., George C., Hussain S.T., Grundy K., Dennett L. (2020). Analgesic use in patients with advanced chronic kidney disease: a systematic review and meta-analysis. Can J Kidney Health Dis.

[13] Ishida J.H., McCulloch C.E., Steinman M.A., Grimes B.A., Johansen K.L. (2018). Opioid analgesics and adverse outcomes among hemodialysis patients. Clin J Am Soc Nephrol.

[14] Jadoul M., Albert J.M., Akiba T. (2006). Incidence and risk factors for hip or other bone fractures among hemodialysis patients in the Dialysis Outcomes and Practice Patterns Study. Kidney Int.

[15] Vangala C., Niu J., Montez-Rath M.E. (2020). Hip fracture risk among hemodialysis-dependent patients prescribed opioids and gabapentinoids. J Am Soc Nephrol.

[16] Kimmel P.L., Fwu C.W., Abbott K.C., Eggers A.W., Kline P.P., Eggers P.W. (2017). Opioid prescription, morbidity, and mortality in United States dialysis patients. J Am Soc Nephrol.

[17] Novick T.K., Surapaneni A., Shin J.I. (2019). Associations of opioid prescriptions with death and hospitalization across the spectrum of estimated GFR. Clin J Am Soc Nephrol.

[18] You A.S., Kalantar-Zadeh K., Streja E. (2020). Mortality risk in chronic kidney disease patients transitioning to dialysis: impact of opiate and non-opiate use. Am J Nephrol.

[19] Lee S.Y., Wang J., Chao C.T., Chien K.L., Huang J.W. (2020). Frailty modifies the association between opioid use and mortality in chronic kidney disease patients with diabetes: a population-based cohort study. Aging (Albany NY).

[20] Abbott K.C., Fwu C.W., Eggers P.W., Eggers A.W., Kline P.P., Kimmel P.L. (2018). Opioid prescription, morbidity, and mortality in US transplant recipients. Transplantation.

[21] Zhan M., Doerfler R.M., Xie D. (2020). Association of opioids and nonsteroidal anti-inflammatory drugs with outcomes in CKD: findings from the CRIC (chronic renal insufficiency cohort) study. Am J Kidney Dis.

[22] Barrantes F., Luan F.L., Kommareddi M. (2013). A history of chronic opioid usage prior to kidney transplantation may be associated with increased mortality risk. Kidney Int Rep.

[23] Pham A., Cummings M., Lindeman C., Drummond N., Williamson T. (2019). Recognizing misclassification bias in research and medical practice. Fam Pract.

[24] Kyriacou D.N., Lewis R.J. (2016). Confounding by indication in clinical research. JAMA.

[25] Fu E.L., van Diepen M., Xu Y. (2021). Pharmacoepidemiology for nephrologists (part 2): Potential biases and how to overcome them. Clin Kidney J.

[26] The University of Dundee (2024). Health Informatics Centre. https://www.dundee.ac.uk/locations/health-informatics-centre.

[27] Womersley J. (1996). The public health uses of the Scottish Community Health Index (CHI). J Public Health Med.

[28] Runesson B., Trevisan M., Iseri K. (2020). Fractures and their sequelae in non-dialysis-dependent chronic kidney disease: the Stockholm CREAtinine Measurement project. Nephrol Dial Transplant.

[29] Schmidt-Mende K., Wettermark B., Andersen M. (2019). Prevalence of renally inappropriate medicines in older people with renal impairment - A cross-sectional register-based study in a large primary care population. Basic Clin Pharmacol Toxicol.

[30] Levey A.S., Stevens L.A., Schmid C.H. (2009). A new equation to estimate glomerular filtration rate. Ann Intern Med.

[31] Levey A.S., Eckardt K.U., Tsukamoto Y. (2005). Definition and classification of chronic kidney disease: a position statement from Kidney Disease: improving Global Outcomes (KDIGO). Kidney Int.

[32] Graul E.L., Stone P.W., Massen G.M. (2023). Determining prescriptions in electronic healthcare record data: methods for development of standardized, reproducible drug codelists. JAMIA Open.

[33] Hébert H.L., Morales D.R., Torrance N., Smith B.H., Colvin L.A. (2022). Assessing the impact of a national clinical guideline for the management of chronic pain on opioid prescribing rates: a controlled interrupted time series analysis. Implement Sci.

[34] Scottish Intercollegiate Guideline Network (2013). https://www.sign.ac.uk/assets/sign136.pdf.

[35] Public Health Scotland (2024). Scottish Index of Multiple Deprivation (SIMD). Geography, population and deprivation support. https://publichealthscotland.scot/resources-and-tools/health-intelligence-and-data-management/geography-population-and-deprivation-support/deprivation/scottish-index-of-multiple-deprivation-simd/.

[36] Han Y., Balkrishnan R., Hirth R.A. (2020). Assessment of prescription analgesic use in older adults with and without chronic kidney disease and outcomes. JAMA Netw Open.

[37] Cooper R., Bunn J.G., Richardson S.J. (2024). Rising to the challenge of defining and operationalising multimorbidity in a UK hospital setting: the ADMISSION research collaborative. Eur Geriatr Med.

[38] Trevisan M., Fu E.L., Xu Y. (2020). Pharmacoepidemiology for nephrologists (part 1): Concept, applications and considerations for study design. Clin Kidney J.

[39] Brar S., Ye F., James M.T. (2018). Association of angiotensin-converting enzyme inhibitor or angiotensin receptor blocker use with outcomes after acute kidney injury. JAMA Intern Med.

[40] Ray W.A. (2003). Evaluating medication effects outside of clinical trials: new-user designs. Am J Epidemiol.

[41] Colhoun H.M., Livingstone S.J., Looker H.C. (2012). Hospitalised hip fracture risk with rosiglitazone and pioglitazone use compared with other glucose-lowering drugs. Diabetologia.

[42] Farran B., McGurnaghan S., Looker H.C. (2017). Modelling cumulative exposure for inference about drug effects in observational studies. Pharmacoepidemiol Drug Saf.

[43] Levin D., Bell S., Sund R. (2015). Pioglitazone and bladder cancer risk: a multipopulation pooled, cumulative exposure analysis. Diabetologia.

[44] Xie D., Yang W., Jepson C. (2017). Statistical methods for modeling time-updated exposures in cohort studies of chronic kidney disease. Clin J Am Soc Nephrol.

[45] Thompson W.A. (1977). On the treatment of grouped observations in life studies. Biometrics.

[46] Tennant P.W.G., Murray E.J., Arnold K.F. (2021). Use of directed acyclic graphs (DAGs) to identify confounders in applied health research: review and recommendations. Int J Epidemiol.

[47] Feeney T., Hartwig F.P., Davies N.M. (2025). How to use directed acyclic graphs: guide for clinical researchers. BMJ.

[48] Austin P.C. (2024). A comparison of variance estimators for logistic regression models estimated using generalized estimating equations (GEE) in the context of observational health services research. Stat Med.

[49] Pan W. (2001). Akaike's information criterion in generalized estimating equations. Biometrics.

[50] R Core Team (2024). https://www.R-project.org.

[51] von Elm E., Altman D.G., Egger M. (2007). Strengthening the Reporting of Observational Studies in Epidemiology (STROBE) statement: guidelines for reporting observational studies. BMJ.

[52] Langan S.M., Schmidt S.A., Wing K. (2018). The reporting of studies conducted using observational routinely collected health data statement for pharmacoepidemiology (RECORD-PE). BMJ.

[53] Muanda F.T., Weir M.A., Ahmadi F. (2022). Higher-dose gabapentinoids and the risk of adverse events in older adults with CKD: a population-based cohort study. Am J Kidney Dis.

[54] Fleet J.L., Dixon S.N., Kuwornu P.J. (2018). Gabapentin dose and the 30-day risk of altered mental status in older adults: a retrospective population-based study. PLoS One.

[55] National Institute of Health and Care Excellence (2021). https://www.nice.org.uk/guidance/ng193.

[56] Scottish Intercollegiate Guidelines Network (2025). https://www.sign.ac.uk/guidelines/management-of-chronic-pain/.

[57] Hill J.C., Whitehurst D.G., Lewis M. (2011). Comparison of stratified primary care management for low back pain with current best practice (STarT Back): a randomised controlled trial. Lancet.

[58] Lamb S.E., Hansen Z., Lall R. (2010). Group cognitive behavioural treatment for low-back pain in primary care: a randomised controlled trial and cost-effectiveness analysis. Lancet.

[59] Cherkin D.C., Sherman K.J., Balderson B.H. (2016). Effect of mindfulness-based stress reduction vs cognitive behavioral therapy or usual care on back pain and functional limitations in adults with chronic low back pain: a randomized clinical trial. JAMA.

[60] Thorsell J., Finnes A., Dahl J. (2011). A comparative study of 2 manual-based self-help interventions, acceptance and commitment therapy and applied relaxation, for persons with chronic pain. Clin J Pain.

[61] Becker W.C., Dorflinger L., Edmond S.N., Islam L., Heapy A.A., Fraenkel L. (2017). Barriers and facilitators to use of non-pharmacological treatments in chronic pain. BMC Fam Pract.

