# Supplementary material for: Long-Term Opioid and Gabapentinoid Related Harms in CKD
Source: Kidney Int Rep. 2026 Sep 17;11(12):107085. doi: 10.1016/j.ekir.2026.107085 (PMC13686002; doi:10.1016/j.ekir.2026.107085)
Supplement: Supplementary file 1 [file mmc1.pdf]

Long-term opioid and gabapentinoid related harms in adults with chronic kidney disease: a time-updated, population-based cohort study

Christopher H. Grant[1], Shona Livingstone[1], Huan Wang[1], Karen N. Barnett[1], Patrick B. Mark[2], Lesley A. Colvin[1], Samira Bell[1]

[1] Division of Population Health and Genomics, School of Medicine, University of Dundee, UK

[2] School of Cardiovascular & Metabolic health, University of Glasgow, UK

Supplemental Material

Table of contents

Supplemental Information S1: Data sources & access ... 4

Supplemental Information S2: Inclusion & exclusion criteria ... 6

Supplemental Information S3: Code list generation & clinician review ... 7

Supplemental Information S4: Prescription duration & permissible gap ... 8

Supplemental Information S5: Discrete time logistic model ... 9

Supplemental Information S6: Direct acyclic graph creation ... 11

Supplemental Information S7: Sensitivity analyses ... 15

Supplemental Table S1: List of relevant opioids & gabapentinoids ... 16

Supplemental Table S2: List of data sources, time windows & relevant codes to determine co-morbidity at baseline ... 17

Supplemental Table S3: List of data sources, relevant codes & supporting references to determine cause-specific secondary outcomes ... 19

Supplemental Table S4: Opioid subtypes among patients with baseline 6m opioid use ... 20

Supplemental Table S5: Gabapentinoid subtypes among patients with baseline 6m gabapentinoid use ... 21

Supplemental Table S6: Opioid & gabapentinoid exposure during follow-up ... 21

Supplemental Table S7: Marginal risk of death and odd ratios by 90-day cumulative opioid exposure (minimally adjusted) ... 22

Supplemental Table S8: Marginal risk of death and odd ratios by 90-day cumulative opioid exposure stratified by sex ... 23

Supplemental Table S9: Association between long-term 90d cumulative gabapentinoid exposure & all-cause mortality (minimally adjusted) ... 24

Supplemental Table S10: Association between long-term 90d cumulative gabapentinoid exposure & all-cause mortality stratified by sex ... 25

Supplemental Table S11: Marginal rate of emergency hospitalisation and adjusted rate ratios by 90-day cumulative opioid exposure stratified by sex ... 26

Supplemental Table S12: Association between long-term 90-day cumulative gabapentinoid exposure & emergency hospitalisation stratified by sex ... 27

Supplemental Table S13: Association between long-term 90-day cumulative opioid exposure & hip fractures ... 29

Supplemental Table S14: Association between long-term 90-day cumulative gabapentinoid exposure & hip fractures ... 30

Supplemental Table S15: Marginal risk of death and odd ratios by 90-day cumulative lipid-lowering therapy ... 31

Supplemental Figure S1: Direct acyclic graph depicting the proposed relationship between chronic opioid exposure and mortality ... 32

Supplemental Figure S2: Direct acyclic graph depicting the proposed relationship between chronic gabapentinoid exposure and mortality 33
Supplemental Figure S3: Marginal risk of death by cumulative opioid exposure in females 34
Supplemental Figure S4: Marginal risk of death by cumulative opioid exposure in males 34
Supplemental Figure S5: Marginal rate of emergency hospitalisation by cumulative opioid exposure in females 35
Supplemental Figure S6: Marginal rate of emergency hospitalisation by cumulative opioid exposure in males 35
Supplementary References 36
RECORD-PE Checklist 39

Supplemental Information S1: Data sources & access

Demography

Demographic data for patients resident in Tayside and Fife health boards is obtained from the Scottish national CHI dataset which is a population register of all patients in NHS Scotland based on a 10-character numeric unique identifier.(S1) This is the current or most recent electronic health record for each person in Scotland aiming for a single record per person.(S2) This provided information on whether a person left Tayside/Fife to register with a different health board GP and were therefore no longer observable.

SMR01

SMR01 provides patient episode level data on hospital inpatient and day case discharges from acute hospital specialities, including demographics, admission type, conditions and diagnoses (coded according to the International Statistical Classification of Diseases and Related Health Problems 10th Revision, ICD-10).(S3) SMR01 provides national coverage of all acute hospitals in Scotland with an estimated completion rate of 99%.(S2) SMR01 data are considered amongst the best available routinely collected health data worldwide in relation to completeness, granularity and potential for data linkage.(S4)

SMR06

The Scottish Cancer Registry (SMR06) was established in 1954 and provides data on all new diagnoses of cancer among Scottish residents including key clinical information, such as date of diagnosis, cancer site (coded by ICD-10 diagnostic code) and histological subtype.(S5) Provisional source records of new cancer diagnoses are collected annually and confirmed as registrations by Public Health Scotland following internal quality assurance standards.(S6)

Laboratory results

These include all inpatient and outpatient laboratory test results from NHS Tayside and Fife from 1992 and 2008 onwards, respectively.(S7) Only biochemistry data, specifically, serum creatinine (sCr), was extracted for use in the eGFR CKD-EPI 2009 creatinine equation.(8)

Prescribing information system

The PIS provides data on all NHS prescriptions prescribed, dispensed and reimbursed within the community setting in Scotland including prescription characteristics (e.g. prescriber, formulation code, strength, quantity etc).(S8, S9) The CHI capture rate is close to 100% for prescribed and dispensed prescription items and the data undergo extensive quality assurance procedures.(S9) Data on all medicines from 2009 to 2024 were extracted excluding topical dermal treatments.

Scottish Care Initiative-Diabetes Collaboration

SCI-DC is a fully integrated shared national electronic patient record that supports the treatment of NHS Scotland patients with diabetes and includes functionality for both primary and secondary care practitioners.(S10) It is estimated to include over 99% of patients with diabetes who reside permanently in Scotland and receives data from all general practices nationwide with a record created for each newly diagnosed person.(S11)

Scottish Renal Registry

The SRR is a national register of patients receiving kidney replacement therapy (KRT) for established kidney failure in Scotland.(S12) The registry was established in 1991 and receives data from all kidney units in Scotland thereby ensuring complete data coverage of the KRT population.(S13) The SRR reports epidemiology and audits against recommended best practice to promote high quality care. The SRR includes data on KRT initiation, modality, clinical and laboratory parameters.(S14)

National Records of Scotland Deaths Data

The NRS deaths data contains detailed information about each person who has died in Scotland since 1974, including date and cause of death.(S15, S16) There is a statutory requirement for all deaths in Scotland to be registered with the NRS within eight days of occurrence.(S17) NRS uses ICD-10 diagnostic codes to create a single primary underlying cause of death and up to nine secondary causes.(S18) Scottish deaths data is considered to be world leading due to a robust and independent review system of cause of death data.(S19) Date of death was determined from the CHI death record and supplemented with the NRS General Registrar Office ICD-10 coded cause of death data.

Data access

Specific variables to determine a cohort of adults with biochemically defined CKD during the study period were initially provided by the HIC to study investigators. Researchers used anonymised unique identifier, date of birth, sex and serum creatinine measurements of the population of Tayside and Fife to determine the study CKD cohort as outlined in Figure 1, main text. Data related to all other variables were subsequently provided by the HIC pertaining to the final study cohort. Researchers performed data cleaning using standard approaches.

Supplemental Information S2: Inclusion & exclusion criteria

The source population consisted of people (of any age) alive and resident in the Tayside or Fife health board regions on 1st January 2009 or 1st January 2014, respectively with at least two serum creatinine tests from database inception until date of extraction. These dates were selected due to the availability of accurate prescribing data in each respective health board (i.e. 2009 Tayside & 2014 Fife). The study population included adults (i.e. aged 18 years or older) with CKD according to the Kidney Disease Improving Global Outcomes (KDIGO) consensus definition(31) using the 2009 CKD-EPI creatinine equation(8) (without adjustment for race) defined by the first occurrence of two consecutive eGFR creatinine

(eGFRcr) readings less than 60ml/min/m^2 and separated by at least 90 days. The second confirmatory eGFR was used as the date of entry into the study population if this occurred during the study period (i.e. 1st January 2010 for Tayside or 1st January 2015 Fife until 1st January 2023). Use of the 2009 CKD-EPI creatinine equation without race/ethnicity represents current best practice in UK populations.(S20, S21)

Incident CKD diagnoses from 2010/2015 onwards were selected to allow a minimum of one year of baseline prescribing data and five years of baseline co-morbidity/multimorbidity assessment for all patients prior to possible cohort entry (i.e. index date). Incident CKD cases until 2023 were selected to allow a minimum follow up period of one year for all patients to outcome ascertainment at study end (i.e. 1st January 2024).

A clinically plausible range of serum creatinine values (i.e. 25 to 1,500µmol/L) was based on clinical expertise and as employed in previous studies.(28, 29) Children and young adults (i.e. less than 18 years at date of serum creatinine test), clinically implausible serum creatinine test values and missing serum creatinine test values were excluded. Patients who died, left Tayside/Fife or received kidney replacement therapy, KRT (i.e. dialysis or kidney transplantation for kidney failure less than one day following CKD diagnosis were excluded.

Supplemental Information S3: Code list generation & clinician review

A recommended approach was followed to determine relevant drug code lists in a standardized, reproducible and transparent manner.(32) A list of opioids for pain relief were determined from the British National Formulary (BNF) chapter 4.7.2 'opioid analgesics'(5, 33)(S22, S23) with additional combination medications found elsewhere (Supplemental Table S1).(33) A list of gabapentinoids (i.e. gabapentin and pregabalin) was determined from BNF chapter 4.8.1 'control of epilepsy'.(5, 33)(S23) Neither gabapentin nor pregabalin were identified from other potentially relevant BNF chapters (e.g. 4.7.3 'neuropathic pain') within our dataset. All drugs (non-proprietary name) were searched semantically in the BNF codelist meta-data file provided by the HIC based on BNF drug name column to generate a list of all possible codes to identify each analgesic of interest. The BNF uses the British Approved Name for specific drugs which is identical to the Recommended International

Non-proprietary Name (rINN) for medicinal substances, as such, brand (i.e. proprietary) drug names (e.g. oramorph) are not employed.(S24)

To ensure complete capture, all drugs in the relevant BNF chapters were screened for applicability. BNF codes pertaining to the list of drugs were then reviewed by a clinician panel (i.e. CHG, LC and PM) with alterations made as necessary to determine a final list of applicable drug codes (i.e. removal of injectables, 'dummy' or 'NA' entries or any non-combination opioid products from outside of 4.7.2 [e.g. 3.9.1 'cough supplements']).(32) Other individual code lines removed were 'buprenorphine and naloxone', 'morphine with cocaine', 'morphine and cocaine' and 'morphine with atropine' as they were deemed to be out with scope following clinician review. Details of clinician code review and list of all relevant final BNF codes, names and relevant chapters used to determine exposure to each drug class are available upon reasonable request to the corresponding author.

Supplemental Information S4: Prescription duration & permissible gap

The duration of each prescription was unknown since the free form entries for the directions for intended use (e.g. 'take two tablets every four hours for seven days') were missing in 70.3% of relevant analgesics.A duration of 28 days was therefore assumed for each prescription from the date of prescription dispensation). This was selected as prescriptions for controlled drugs are valid for 28 days and maximal monthly supply is considered best practice.(S25-S27) Cumulative exposure was defined using the refill-gap method based on the dispensed prescription date, assumed duration (i.e. 28 days), projected end date (i.e. dispensed prescription date plus 28 days) and permissible gap (i.e. 28 days).(28, 29) For any two consecutive prescriptions, if the second prescription date occurred within 28 days or fewer from the projected end date of the preceding prescription, the patient was judged to have continued the analgesic.(S28) This permissible gap was judged to be indicative of late prescription refills or stockpiling rather than non-adherence or cessation.(S29) If the second prescription date occurred > 28 days after the estimated end date of the first then the patient was judged to have stopped taking the analgesic from day 29. The permissible gap was selected to ensure alignment with our primary exposure

(i.e. 90 days of analgesic exposure per 180 days) consistent with prior work.(S26, S28) Patients with only a single prescription (i.e. 28 days of assumed analgesic exposure) did not meet the threshold for primary exposure classification.

The assumed duration and permissible gap were varied in our sensitivity analyses (see Supplemental Information S7: Sensitivity Analyses).

Supplemental Information S5: Discrete time logistic model

Data was formatted for model input as follows. The primary outcome was all-cause mortality as determined by NRS. Patients were followed from CKD diagnosis (i.e. study entry) until the earliest of date of exit from Tayside/Fife health board, death or study end (i.e. 1st January 2024) with patient follow-up separated into sequential 28-day time intervals from entry to exit. Only the final time interval could be less than 28-days when right censored due to exit from health board, death or study end. A data table was created with one row per 28-day interval per individual each labelled with a binary indicator for death by the end of the interval, and patient status at the start of each interval (time t) recorded for time-fixed covariates (i.e. sex, SIMD) and time updated covariates (i.e. age, eGFRcr, years since CKD diagnosis, co-morbid conditions, ever exposed to opioids or gabapentinoids and cumulative 90-day analgesic exposure). Time updated covariate status was determined cumulatively from baseline (see Supplemental Table S2) until the start of each interval, time t. Age at time t was derived from anonymised date of birth. eGFRcr was derived from the most recent serum creatinine blood test prior to time t. Years since CKD diagnosis was derived as the difference in years between CKD diagnosis date (i.e. study entry) and time t. Co-morbid conditions were derived as outlined in Supplemental Table S2 carried forward from baseline to time t. Ever exposure to opioids or gabapentinoids were established from any single prescription to either relevant drug class from study entry until time t.

A multivariable, discrete time logistic model of mortality was fitted to this dataset to estimate the odds of death within each 28 day interval and assess the impact of cumulative analgesic exposure on mortality risk among new analgesic users, with each individual

contributing one observation for each 28-day interval between their entry and exit dates. These methods have been employed previously.(41, 45, 42, 43)

Comparison between ever and never analgesic users is likely to be confounded by indication given that these groups of patients are materially different. This between-person confounding can be addressed by including two analgesic exposure terms within the model: the first, a time-updated ever-exposure term until the beginning of each 28 day time period and the second, a time-updated cumulative exposure term.(42) The former accounts for the confounding effect of time-invariant frailty or treatment allocation bias as it contrasts the rate in unexposed person time-intervals with the predicted rate in person time-intervals when exposure has just started. The time-updated ever term is the sum of any immediate effect of drug exposure on mortality and the confounding effect of time-fixed allocation bias. Inclusion of the ever term means that the likelihood of the cumulative effect term does not depend on events in the unexposed and hence cannot be influenced by allocation bias. Interpretation of drug harms is thus focused on the cumulative 90 day exposure term.

To reduce the problem of predicted probabilities close to 0 or 1, continuous variables were centred before fitting to the model. An interaction term was created between age and CKD duration terms given that both measure time since entry. SIMD quintiles were grouped into 1-2 (most deprived), 3 (median) and 4-5 (least deprived) for comparison. The final model included time dependent terms for duration of CKD diagnosis, age, an interaction term (age x CKD duration), eGFRcr, cardiovascular disease, cancer, depression, respiratory disease, diabetes and ever prior analgesic use and baseline sex and socioeconomic status. Models were also compared when stratified by sex.

Generalized estimating equations (GEE) with a binomial family and logistic regression modelling was performed to account for correlation between repeated measures within individuals. GEE provides a conservative estimate of the population average effect of an exposure on a binary outcome when handling clustered data.(48) An exchangeable working correlation was applied.(30) A non-linear association between cumulative analgesic use and adverse outcomes was considered clinically plausible hence analgesic exposure terms were modelled using linear and quadratic (i.e. linear and squared) terms with the quasi-likelihood under the independence model criterion (QIC) compared amongst each exposure-outcome pair.(44) Where quadratic terms were selected due to improved model fit (i.e. cumulative

opioids and mortality or emergency hospitalisation), marginal standardisation was used to present absolute and relative risks across exposure levels (i.e. one to six vs zero 90 day cumulative exposures). For each exposure level, the covariate pattern for all patients was refitted with the exposure set at a pre-specified level (i.e population mean). Predicted log-odds were derived from the fitted GEE model and predicted probabilities converted using the inverse-logit function then averaged across all patients to obtain population-averaged risks with 95% confidence intervals derived from the model's variance-covariance matrix.(S31) Model diagnostics were checked including convergence, cluster structure, co-efficient and standard error stability, ranges of fitted values and predicted risk ranges along with binned residual plots.

Secondary outcomes were analysed using the same approach, however, Poisson regression was applied to emergency hospitalisation as a frequent and potentially recurrent event (i.e. within any 28 day time interval) with an offset term included to allow for varying follow-up time across intervals.

Supplemental Information S6: Direct acyclic graph creation

To inform the analyses and determine a sufficient adjustment set for the exposure outcome relationship of interest, direct acyclic graphs (DAG) were created.(46, 47) DAGs make explicit the assumptions made by researchers in assessing causal pathways in observational research including variables considered to be confounders, mediators and colliders.(47) These DAG diagrams were developed a priori to represent the presumed causal relationship between chronic analgesic exposure and mortality. Each DAG was drafted by the lead author (CHG) based on subject-matter knowledge, previous literature (36) and data availability with review by senior authors with clinical expertise (PBM, LAC and SB). Nodes selected were those judged to be the most important factors influencing analgesic prescription, mortality and the relationship between exposure and outcome, including potential mediators and colliders. Directed edges represented hypothesised causal effects and based on clinical and epidemiological consensus. The DAGs for impact of long-term opioids and gabapentinoids on mortality are displayed in Supplemental Figures S1 & S2. These graphical depictions,

created using the DAGitty online browser platform (https://www.dagitty.net/), summarise the assumed relevant direct and indirect causal and confounding pathways between chronic analgesic use and death.(S32) The code used to create each DAG is appended below for reproducibility. Each DAG highlighted the need for a set of variables required to adjust for potential bias to estimate the total effect of chronic analgesia on death as follows; eGFRcr, age, cardiovascular disease, cancer, depression, respiratory disease, diabetes, sex and socioeconomic status (i.e. fully adjusted main models). Minimally adjusted (i.e. age and sex) models are presented for the primary outcome for comparison (Supplemental Table S7 & S9).

Exposure: chronic opioids. Outcome: all-cause mortality

```
dag {
"Chronic pain" [latent,pos="-0.720,1.563"]
"Liver disease" [pos="-1.023,0.188"]
"Opioid use disorder" [pos="-0.478,-0.306"]
"Respiratory disease" [pos="-0.620,0.717"]
"Rheumatoid arthritis" [pos="-0.853,0.826"]
Age [pos="-1.439,-0.621"]
CVD [pos="-0.615,0.277"]
Cancer [pos="-0.865,-0.158"]
Death [outcome,pos="0.048,0.330"]
Depression [pos="-1.033,1.440"]
Deprivation [pos="-1.477,0.418"]
Diabetes [pos="-1.035,0.697"]
Opioids [exposure,pos="-0.469,0.991"]
Sex [pos="-1.483,-0.152"]
eGFR [pos="-0.625,-0.627"]
"Chronic pain" -> Opioids
"Liver disease" -> CVD
"Liver disease" -> Cancer
"Liver disease" -> Diabetes
"Opioid use disorder" -> Death
"Respiratory disease" -> "Chronic pain"
"Respiratory disease" -> Cancer
"Respiratory disease" -> Death
"Respiratory disease" -> Depression
"Respiratory disease" -> Opioids
"Rheumatoid arthritis" -> "Chronic pain"
"Rheumatoid arthritis" -> "Respiratory disease"
"Rheumatoid arthritis" -> CVD
"Rheumatoid arthritis" -> Cancer
"Rheumatoid arthritis" -> Depression
Age -> "Chronic pain"
Age -> "Liver disease"
Age -> "Respiratory disease"
Age -> "Rheumatoid arthritis"
Age -> CVD
Age -> Cancer
Age -> Death
```

```
Age -> Diabetes
Age -> eGFR
CVD -> Death
CVD -> Depression
Cancer -> "Chronic pain"
Cancer -> Death
Cancer -> Depression
Depression -> "Chronic pain"
Depression -> Death
Deprivation -> "Chronic pain"
Deprivation -> "Liver disease"
Deprivation -> "Opioid use disorder"
Deprivation -> "Respiratory disease"
Deprivation -> CVD
Deprivation -> Death
Deprivation -> Depression
Deprivation -> Diabetes
Deprivation -> eGFR
Diabetes -> "Chronic pain"
Diabetes -> CVD
Diabetes -> Death
Diabetes -> eGFR
Opioids -> "Opioid use disorder"
Opioids -> Death
Sex -> "Chronic pain"
Sex -> "Opioid use disorder"
Sex -> "Respiratory disease"
Sex -> "Rheumatoid arthritis"
Sex -> CVD
Sex -> Cancer
Sex -> Death
Sex -> Depression
Sex -> eGFR
eGFR -> "Chronic pain"
eGFR -> CVD
eGFR -> Cancer
eGFR -> Death
eGFR -> Depression
}
```

Exposure: chronic gabapentinoids. Outcome: all-cause mortality

```
dag {
"Chronic pain" [latent,pos="-0.720,1.563"]
"Liver disease" [pos="-1.023,0.188"]
"Respiratory disease" [pos="-0.620,0.717"]
"Rheumatoid arthritis" [pos="-0.853,0.826"]
Age [pos="-1.439,-0.621"]
CVD [pos="-0.615,0.277"]
Cancer [pos="-0.865,-0.158"]
Death [outcome,pos="0.048,0.330"]
Depression [pos="-1.033,1.440"]
Deprivation [pos="-1.477,0.418"]
Diabetes [pos="-1.035,0.697"]
Gabapentinoids [exposure,pos="-0.478,1.132"]
Sex [pos="-1.483,-0.152"]
```

```
eGFR [pos="-0.625,-0.627"]
"Chronic pain" -> Gabapentinoids
"Liver disease" -> CVD
"Liver disease" -> Cancer
"Liver disease" -> Diabetes
"Respiratory disease" -> "Chronic pain"
"Respiratory disease" -> Cancer
"Respiratory disease" -> Death
"Respiratory disease" -> Depression
"Rheumatoid arthritis" -> "Chronic pain"
"Rheumatoid arthritis" -> "Respiratory disease"
"Rheumatoid arthritis" -> CVD
"Rheumatoid arthritis" -> Cancer
"Rheumatoid arthritis" -> Depression
Age -> "Chronic pain"
Age -> "Liver disease"
Age -> "Respiratory disease"
Age -> "Rheumatoid arthritis"
Age -> CVD
Age -> Cancer
Age -> Death
Age -> Diabetes
Age -> eGFR
CVD -> Death
CVD -> Depression
Cancer -> "Chronic pain"
Cancer -> Death
Cancer -> Depression
Depression -> "Chronic pain"
Depression -> Death
Depression -> Gabapentinoids
Deprivation -> "Chronic pain"
Deprivation -> "Liver disease"
Deprivation -> "Respiratory disease"
Deprivation -> CVD
Deprivation -> Death
Deprivation -> Depression
Deprivation -> Diabetes
Deprivation -> eGFR
Diabetes -> "Chronic pain"
Diabetes -> CVD
Diabetes -> Death
Diabetes -> eGFR
Gabapentinoids -> Death
Sex -> "Chronic pain"
Sex -> "Respiratory disease"
Sex -> "Rheumatoid arthritis"
Sex -> CVD
Sex -> Cancer
Sex -> Death
Sex -> Depression
Sex -> eGFR
eGFR -> "Chronic pain"
eGFR -> CVD
eGFR -> Cancer
eGFR -> Death
eGFR -> Depression
```

Supplemental Information S7: Sensitivity analyses

Multiple sensitivity analyses were performed. Firstly, the requirement for a second confirmatory eGFR at 90 days to determine CKD status potentially under-estimates CKD prevalence hence, the analysis was re-run in a renal dysfunction cohort of patients with two consecutive eGFR < 60ml/min/m^2 separated by one day.(S33) Secondly, to test whether the exclusion of patients based on a single, short-term analgesic prescription altered the observed results, only established prevalent users (i.e. one prescription in six months plus one 90-day stretch of analgesic use in one year prior to study entry) were excluded. Thirdly, both the assumed duration and permissible gap were varied to 56 and 14 days, respectively. Finally, lipid-lowering therapies (i.e. BNF 2.12) were selected as a negative control exposure as they share similar confounders, but none of the same direct biological causal paths between opioids/gabapentinoids and death.(S34)

Supplemental Table S1: List of relevant opioids & gabapentinoids

Drug	Type	Strength
Opioids		
Co-codamol	Combination	Weak
Tramadol hydrochloride	Single	Weak
Co-dydramol	Combination	Weak
Dihydrocodeine	Single	Weak
Methadone	Single	Strong
Morphine	Single	Strong
Oxycodone	Single	Strong
Codeine	Single	Weak
Fentanyl	Single	Strong
Buprenorphine	Single	Strong
Co-codamol with buclizine	Combination	Weak
Paracetamol with tramadol	Combination	Weak
Diamorphine	Single	Strong
Meptazinol	Single	Weak
Tapentadol	Single	Strong
Pethidine	Single	Strong
Hydromorphone hydrochloride	Single	Strong
Dipipanone with cyclizine	Combination	Strong
Oxycodone and naloxone	Combination	Strong
Pentazocine	Single	Strong
Co-codaprin	Combination	Weak
Ibuprofen with codeine phosphate	Combination	Weak
Aspirin with papaveretum	Combination	Strong
Paracetamol, codeine & caffeine	Combination	Weak
Gabapentinoids		
Gabapentin	Single	n/a
Pregabalin	Single	n/a

Supplemental Table S2: List of data sources, time windows & relevant codes to determine co-morbidity at baseline

Condition	Data source(s), time window & relevant codes
**Cardiovascular disease**<br>• *Aneurysm, arrhythmia, coronary artery disease, heart failure, heart valve disorders, hypertension, peripheral artery disease, stroke or venous thromboembolism*	*SMR01 (5y)*<br>**ICD-10**: I710–I716, I718–I719, I442, I460, I470–I472, I48X, I480–I484, I489, I495, Z950, I200–I201, I208–I209, I210–I214, I219, I220–I221, I228–I229, I230–I236, I238, I241, I250–I256, I258–I259, I110, I130, I132, I500–I501, I509, I050–I052, I058–I059, I060–I062, I068–I069, I070–I072, I078–I079, I080–I083, I088–I089, I340–I342, I348–I349, I350–I352, I358–I359, I360–I362, I368–I369, I370–I372, I378–I379, I10X, I110, I119, I120, I129, I130–I132, I139, I150–I152, I158–I159, I702, I7020–I7021, I731, I738–I739, I743–I745, G460–G468, I600–I609, I610–I616, I618–I619, I630–I635, I638–I639, I64X, I260, I269, I801–I803, I81X, I820, I822–I823, I828–I829
**Diabetes mellitus**	*SMR01 (5y)*<br>**ICD-10**: E100, E101, E102, E103, E104, E105, E106, E107, E108, E109, E10X, E110, E111, E112, E113, E114, E115, E116, E117, E118, E119, E11X, E120, E121, E122, E123, E124, E125, E126, E127, E128, E129, E12X, E130, E131, E132, E133, E133D, E134, E135, E136, E137, E138, E139, E13X, E140, E141, E142, E143, E144, E145, E146, E147, E148, E149, E14X, G590, G632, H280, H360, M142, N083<br><br>*PIS (1y)*<br>**BNF**: 060101, 060102<br><br>*SCI-DC (1y)*<br>Type 1, type 2 or diabetes mellitus type not defined
**Cancer**<br>• *Haematological malignancy, melanoma, metastatic malignancy, solid organ cancer*	*SMR01 (5y) / SMR06 (1y)*<br>ICD-10: C810–C814, C817, C819–C829, C830–C831, C833, C835, C837–C839, C840–C841, C844–C849, C851–C852, C857, C859, C860–C866, C880, C882, C900–C903, C910–C919, C920–C929, C930–C931, C933, C937, C939–C947, C950–C951, C957, C959–C969, C430–C439, C440–C449, C450–C459, C770–C775, C778–C799, C800, C809, C000–C009, C01X, C020–C024, C028–C029, C030–C031, C039, C040–C041, C048–C049, C150–C155, C158–C159, C160–C169, C170–C173, C178–C179, C180–C189, C19X–C20X, C210–C212, C218, C220–C224, C227, C229, C23X, C240–C249, C250–C259, C260–C269, C300–C301, C310–C313, C318–C319, C320–C323, C328–C329, C33X, C340–C343, C348–C349, C37X, C380–C384, C388, C390, C398–C399, C400–C419, C460–C469, C470–C479, C480–C488, C490–C499, C500–C509, C510–C519, C52X, C530–C539, C540–C549, C55X, C56X, C570–C579, C58X, C600–C609, C61X,

	C620–C639, C64X–C66X, C670–C679, C680–C689, C690–C699, C700–C719, C720–C729, C73X–C759, C760–C768, C97X
**Depression**	_SMR01 (5y)_<br>**ICD-10**: F320, F321, F322, F323, F328, F329, F330, F331, F332, F333, F334, F338, F339, F412<br><br>_PIS (1y)_<br>**BNF**: 0403
**Chronic liver disease**	_SMR01 (5y)_<br>**ICD-10**: B180, B181, B182, B188, B189, K700, K701, K702, K703, K704, K709, K717, K721, K730, K731, K732, K738, K740, K741, K742, K743, K744, K745, K746, K754, K758, K760, K766, T864
**Chronic respiratory disease**<br>• *Asthma, chronic obstructive pulmonary disease*	_SMR01 (5y)_<br>**ICD-10**: J450, J451, J458, J459, J46X; J40X, J410, J411, J418, J42X, J430, J431, J432, J438, J439, J440, J441, J448, J449<br><br>_PIS (1y)_<br>**BNF**: 030101, 030102, 030104, 0302, 030302
**Rheumatoid arthritis**	_SMR01 (5y)_<br>**ICD-10**: M053, M0530–M0539, M058, M0580–M0589, M059, M0590–M0599, M060, M0600–M0609, M068, M0680–M0689, M069, M0690–M0699, M080, M0800–M0809
**Opioid use disorder**	_PIS (1y)_<br>**BNF**: 04103

The look-back windows for each data source from index date (i.e. CKD diagnosis) was as follows: smr01 5 years, PIS 1 year, smr06 1 year, sci-DC 1 year. For PIS defined indicators, the presence of a single BNF code in prescription data in the 365 days preceding index was sufficient to identify comorbidity. Abbreviations: BNF: British National Formulary; DM: diabetes mellitus. ICD: international classification of diseases, PIS: prescribing information system., SMR01: Scottish morbidity record 01; SMR06 Scottish Cancer registry; sci-DC the SCI-Diabetes collaboration. ICD-10 code lists for relevant conditions adapted from *Cooper* et al.(37)

Supplemental Table S3: List of data sources, relevant codes & supporting references to determine cause-specific secondary outcomes

Condition	Data source(s) & relevant codes
**Emergency hospitalization** [S35, S36]<br>• *All-cause emergency hospitalization*	*SMR01*<br>Admission type: 30: Emergency Admission; 31-35: Patient Injury; 36: Patient Non-Injury (e.g. stroke, MI, Ruptured Appendix); 38: Other Emergency Admission (including emergency transfers); 39 Emergency Admission, type not known
**Hip fracture** [S37]<br>• *Emergency hospitalization for femoral neck, intertrochanteric or subtrochanteric fracture*	*SMR01*<br>ICD-10 [£]: s720, s721, s722

[£] Secondary outcomes were ascertained based on the first two diagnostic column from the SMR01 record. Abbreviations: ICD: international classification of diseases, PIS: prescribing information system, SMR01: Scottish morbidity record 01, SRR: Scottish Renal Registry.

Supplemental Table S4: Opioid subtypes among patients with baseline 6m opioid use

Analgesic	n	Percentage (%)
Co-codamol	4,055	47.7
Tramadol	2,075	24.4
Codeine	1,236	14.5
Morphine	765	9.0
Codydramol	681	8.0
Dihydrocodeine	410	4.8
Oxycodone	406	4.8
Fentanyl	152	1.8
Buprenorphine	110	1.3
Cocodamol Buclizine	30	0.4
Meptazinol	21	0.2
Paracetamol Tramadol	7	0.1
Tapentadol	7	0.1
Opioids	8,508	100.0

Note: 8,508 patients with baseline opioid use (i.e. any single prescription six months prior to CKD diagnosis). Opioid subtypes are not mutually exclusive. Hydromorphone, pethidine, methadone & oxycodone/naloxone were all n<5 (0.0%) hence not displayed.

Supplemental Table S5: Gabapentinoid subtypes among patients with baseline 6m gabapentinoid use

Analgesic	n	Percentage (%)
Gabapentin	1,273	65.3
Pregabalin	742	38.1
Gabapentinoids	1,949	100.0

Note: Percentages based on n = 1,949 patients with baseline gabapentinoid use (i.e. any prescription within six months prior to CKD diagnosis). Gabapentinoid subtypes are not mutually exclusive.

Supplemental Table S6: Opioid & gabapentinoid exposure during follow-up

	Opioids	Gabapentinoids
Number of patients with any prescription *	10,328 (48.2)	1,088 (5.1)
Median number of prescriptions £	2 (1-6)	2 (1-9)
Median days exposed £	57 (28-161)	56 (28-197)
≥ One 90-day cumulative exposure	2,399 (11.2)	350 (1.6)
Median number of 90-day cumulative exposures $	2 (1-5)	4 (1-9)

Percentages based on n=21,440 non-prevalent opioid or gabapentinoid users with non-missing SIMD at baseline. Columns refer to number (%) or median (IQR) as relevant. * i.e. one or more prescription following study entry until censor. £ among those with at least one or more prescription following study entry. $ among those with at least one 90-day cumulative exposure following study entry

Supplemental Table S7: Marginal risk of death and odd ratios by 90-day cumulative opioid exposure (minimally adjusted)

90-day continuous opioid stretches	Risk of death (95% CI)	Odds ratio (95% CI)	P-value
0	0.4 (0.4-0.4)	1.00 (1.00–1.00)	NA
1	0.5 (0.5-0.5)	1.20 (1.16-1.24)	< 0.001
2	0.6 (0.5-0.6)	1.44 (1.35-1.55)	< 0.001
3	0.7 (0.6-0.8)	1.73 (1.56-1.92)	< 0.001
4	0.8 (0.7-1.0)	2.08 (1.81-2.39)	< 0.001
5	1.0 (0.9-1.2)	2.50 (2.10-2.97)	< 0.001
6	1.2 (1.0-1.5)	3.00 (2.44-3.69)	< 0.001

Based on n=21,440 non-prevalent opioid or gabapentinoid users at baseline without missing SIMD. Probability of death during the 28-day time interval having survived to the start of that interval derived from a GEE logistic model including the following terms: cumulative 90 day opioid use (linear), cumulative 90 day opioid use (quadratic), current age (years, centred, linear) and baseline sex (fixed). OR are relative to an exposure of 0. Covariates time-updated unless otherwise specified. P values are Wald tests comparing each opioid exposure level with the reference level derived from the fitted GEE model.

Supplemental Table S8: Marginal risk of death and odd ratios by 90-day cumulative opioid exposure stratified by sex

	Males			Females		
90-day opioid stretches	Risk of death %	aOR (95% CI)	P-value	Risk of death %	aOR (95% CI)	P-value
0	0.4 (0.3–0.4)	1.00 (1.00–1.00)	NA	0.3 (0.3–0.3)	1.00 (1.00–1.00)	NA
1	0.4 (0.4–0.4)	1.13 (1.07–1.18)	< 0.001	0.3 (0.3–0.3)	1.07 (1.04–1.11)	< 0.001
2	0.5 (0.4–0.5)	1.27 (1.15–1.40)	< 0.001	0.3 (0.3–0.4)	1.15 (1.07–1.23)	< 0.001
3	0.5 (0.4–0.6)	1.43 (1.23–1.66)	< 0.001	0.4 (0.3–0.4)	1.23 (1.11–1.37)	< 0.001
4	0.6 (0.5–0.7)	1.61 (1.32–1.97)	< 0.001	0.4 (0.3–0.4)	1.32 (1.15–1.52)	< 0.001
5	0.7 (0.5–0.8)	1.82 (1.41–2.34)	< 0.001	0.4 (0.4–0.5)	1.42 (1.19–1.68)	< 0.001
6	0.7 (0.6–1.0)	2.05 (1.51–2.77)	< 0.001	0.4 (0.4–0.5)	1.52 (1.23–1.87)	< 0.001

Based on n=21,440 non-prevalent opioid or gabapentinoid users at baseline without missing SIMD. Probability of death during the 28-day time interval having survived to the start of that interval derived from a GEE logistic model including the following terms: cumulative 90 day opioid use (linear), cumulative 90 day opioid use (quadratic), duration of CKD diagnosis (years, centred, linear), current age (years, centred, linear), age x CKD duration interaction, eGFRcr (linear), cardiovascular disease, cancer, depression, respiratory disease, diabetes, ever prior opioid use, ever prior gabapentinoid use and socioeconomic status (fixed). Model is stratified by baseline sex (fixed). OR are relative to an exposure of 0. Covariates time-updated unless otherwise specified. P values are Wald tests comparing each opioid exposure level with the reference level derived from the fitted GEE model.

Supplemental Table S9: Association between long-term 90d cumulative gabapentinoid exposure & all-cause mortality (minimally adjusted)

	**Odds ratio (95% CI)**	**P-value**
Age (current, years)	1.09 (1.08-1.09)	<0.001
Female sex	0.77 (0.74-0.81)	<0.001
Cumulative 90d gabapentinoid use	1.05 (1.02-1.07)	<0.001

Based on n=21,440 non-prevalent opioid or gabapentinoid users at baseline without missing SIMD. GEE logistic model including the following terms: cumulative 90 day gabapentinoid use (linear), current age (years, centred, linear) and baseline sex (fixed). Estimates shown are odds ratios with Wald 95% CIs and p-values. Each variable is time-updated unless otherwise specified.

Supplemental Table S10: Association between long-term 90d cumulative gabapentinoid exposure & all-cause mortality stratified by sex

	Males		Females	
**Covariate**	**aOR (95% CI)**	**p-value**	**aOR (95% CI)**	**p-value**
Age (current, years)	1.08 (1.08–1.09)	<0.001	1.09 (1.08–1.10)	<0.001
Age x CKD duration interaction	1.00 (1.00–1.00)	0.008	1.00 (1.00–1.00)	0.011
CKD duration (years)	0.89 (0.87–0.91)	<0.001	0.91 (0.89–0.93)	<0.001
Cancer	2.25 (2.09–2.41)	<0.001	2.48 (2.27–2.71)	<0.001
Cardiovascular disease	2.07 (1.93–2.23)	<0.001	2.06 (1.91–2.21)	<0.001
Chronic respiratory disease	1.44 (1.33–1.54)	<0.001	1.30 (1.20–1.42)	<0.001
Cumulative 90d gabapentinoid use	1.04 (1.00–1.08)	0.050	1.02 (0.99–1.05)	0.201
Depression	1.61 (1.49–1.73)	<0.001	1.43 (1.33–1.54)	<0.001
Diabetes mellitus	1.18 (1.09–1.27)	<0.001	1.23 (1.13–1.34)	<0.001
Gabapentinoid ever use	1.02 (0.85–1.22)	0.862	0.79 (0.66–0.95)	0.012
Opioid ever use	1.61 (1.50–1.73)	<0.001	1.61 (1.49–1.74)	<0.001
SIMD 3 (baseline)	0.90 (0.82–0.99)	0.029	0.94 (0.85–1.05)	0.265
SIMD 4-5 (baseline)	0.85 (0.79–0.92)	<0.001	0.87 (0.80–0.94)	<0.001
eGFR creatinine	0.99 (0.98–0.99)	<0.001	0.99 (0.99–1.00)	<0.001

Based on n=21,440 non-prevalent opioid or gabapentinoid users at baseline without missing SIMD. GEE logistic model including the following terms: cumulative 90 day gabapentinoid use (linear), duration of CKD diagnosis (years, centred, linear), current age (years, centred, linear), age x CKD duration interaction, eGFRcr (linear), cardiovascular disease, cancer, depression, respiratory disease, diabetes, ever prior opioid use, ever prior gabapentinoid use and socioeconomic status (fixed). Model is stratified by baseline sex (fixed). Estimates shown are odds ratios with Wald 95% CIs and p-values. Each variable is time-updated unless otherwise specified.

Supplemental Table S11: Marginal rate of emergency hospitalisation and adjusted rate ratios by 90-day cumulative opioid exposure stratified by sex

	Males			Females		
90-day opioid stretches	Emergency hospitalisation	aRR (95% CI)	P-value	Emergency hospitalisation	aRR (95% CI)	P-value
0	36.42 (35.79–37.06)	1.00 (1.00–1.00)	NA	29.67 (28.71–30.65)	1.00 (1.00–1.00)	NA
1	38.37 (37.62–39.14)	1.05 (1.04–1.07)	< 0.001	30.30 (29.26–31.38)	1.02 (1.00–1.04)	0.006
2	40.43 (39.25–41.63)	1.11 (1.08–1.14)	< 0.001	30.95 (29.54–32.42)	1.04 (1.00–1.09)	0.005
3	42.60 (40.86–44.40)	1.17 (1.12–1.22)	< 0.001	31.61 (29.70–33.64)	1.07 (1.00–1.13)	0.004
4	44.88 (42.50–47.40)	1.23 (1.17–1.30)	< 0.001	32.29 (29.81–34.97)	1.09 (1.01–1.18)	0.002
5	47.29 (44.18–50.61)	1.30 (1.21–1.39)	< 0.001	32.98 (29.89–36.38)	1.11 (1.01–1.23)	0.002
6	49.82 (45.93–54.05)	1.37 (1.26–1.49)	< 0.001	33.68 (29.96–37.86)	1.14 (1.01–1.28)	< 0.001

Based on n=21,440 non-prevalent opioid or gabapentinoid users at baseline without missing SIMD. Event rate during the 28-day time interval having survived to the start of that interval derived from a GEE Poisson model including the following terms: cumulative 90 day opioid use (linear), cumulative 90 day opioid use (quadratic), duration of CKD diagnosis (years, centred, linear), current age (years, centred, linear), age x CKD duration interaction, eGFRcr (linear), cardiovascular disease, cancer, depression, respiratory disease, diabetes, ever prior opioid use, ever prior gabapentinoid use and socioeconomic status (fixed). Model is stratified by baseline sex (fixed). RR are relative to an exposure of 0. Covariates time-updated unless otherwise specified. P values are Wald tests of the rate-ratio contrast comparing each opioid exposure level with the reference level derived from the fitted GEE model.

Supplemental Table S12: Association between long-term 90-day cumulative gabapentinoid exposure & emergency hospitalisation stratified by sex

	Men		*Women*	
**Covariate**	**Rate ratio (95% CI)**	**p-value**	**Rate ratio (95% CI)**	**p-value**
Age (current, years)	1.03 (1.03–1.03)	<0.001	1.03 (1.03–1.03)	<0.001
Age x CKD duration interaction	1.00 (1.00–1.00)	<0.001	1.00 (1.00–1.00)	<0.001
CKD duration (years)	0.95 (0.94–0.95)	<0.001	0.95 (0.94–0.96)	<0.001
Cancer	1.60 (1.51–1.68)	<0.001	1.69 (1.59–1.81)	<0.001
Cardiovascular disease	1.65 (1.57–1.74)	<0.001	1.65 (1.56–1.74)	<0.001
Chronic respiratory disease	1.30 (1.23–1.37)	<0.001	1.36 (1.28–1.44)	<0.001
Cumulative 90d gabapentinoid exposure	1.03 (1.00–1.05)	0.036	1.01 (0.99–1.03)	0.214
Depression	1.30 (1.23–1.37)	<0.001	1.26 (1.20–1.33)	<0.001
Diabetes mellitus	1.21 (1.15–1.28)	<0.001	1.22 (1.15–1.30)	<0.001
Gabapentinoid ever use	1.21 (1.07–1.35)	0.001	0.98 (0.87–1.10)	0.721
Opioid ever use	1.27 (1.22–1.33)	<0.001	1.21 (1.16–1.28)	<0.001
SIMD 3 (baseline)	0.91 (0.84–0.98)	0.008	0.91 (0.84–0.98)	0.011
SIMD 4-5 (baseline)	0.85 (0.80–0.90)	<0.001	0.89 (0.84–0.95)	<0.001
eGFR creatinine	0.98 (0.98–0.98)	<0.001	0.98 (0.98–0.99)	<0.001

Based on n=21,440 non-prevalent opioid or gabapentinoid users at baseline without missing SIMD. Event rate during the 28-day time interval having survived to the start of that interval derived from a GEE Poisson model including the following terms: cumulative 90 day gabapentinoid use (linear), duration of CKD diagnosis (years, centred, linear), current age (years, centred, linear), age x CKD duration interaction,

eGFRcr (linear), cardiovascular disease, cancer, depression, respiratory disease, diabetes, ever prior opioid use, ever prior gabapentinoid use and socioeconomic status (fixed). Model is stratified by baseline sex (fixed). RR are relative to an exposure of 0. Covariates time-updated unless otherwise specified.

Supplemental Table S13: Association between long-term 90-day cumulative opioid exposure & hip fractures

Covariate	Odds ratio (95% CI)	p-value
Age (current, years)	1.10 (1.09–1.12)	<0.001
Female sex	1.75 (1.50–2.05)	<0.001
CKD duration (years)	1.01 (0.97–1.05)	0.809
Age x CKD duration interaction	1.00 (0.99–1.00)	0.078
SIMD 3 (baseline)	1.08 (0.88–1.32)	0.478
SIMD 4-5 (baseline)	1.02 (0.86–1.21)	0.830
Cardiovascular disease	1.32 (1.13–1.53)	<0.001
Cancer	1.01 (0.84–1.23)	0.891
Depression	1.57 (1.34–1.83)	<0.001
Diabetes mellitus	0.97 (0.81–1.18)	0.778
Chronic respiratory disease	1.16 (0.98–1.37)	0.090
eGFR creatinine	1.00 (0.99–1.01)	0.858
Gabapentinoid ever use	1.00 (0.69–1.43)	0.977
Opioid ever use	1.03 (0.88–1.21)	0.679
Cumulative 90d opioid exposure	0.97 (0.95–1.03)	0.489

Based on n=21,440 non-prevalent opioid or gabapentinoid users at baseline without missing SIMD. GEE logistic model including the following terms: cumulative 90 day opioid use (linear), duration of CKD diagnosis (years, centred, linear), current age (years, centred, linear), age x CKD duration interaction, eGFRcr (linear), cardiovascular disease, cancer, depression, respiratory disease, diabetes, ever prior opioid use, ever prior gabapentinoid use and baseline sex (fixed) and socioeconomic status (fixed). Estimates shown are odds ratios with Wald 95% CIs and p-values. Each variable is time-updated unless otherwise specified.

Supplemental Table S14: Association between long-term 90-day cumulative gabapentinoid exposure & hip fractures

Covariate	Odds ratio (95% CI)	p-value
Age (current, years)	1.10 (1.09–1.12)	<0.001
Female sex	1.75 (1.50–2.05)	<0.001
CKD duration (years)	1.00 (0.97–1.05)	0.829
Age x CKD duration interaction	1.00 (0.99–1.00)	0.079
SIMD 3 (baseline)	1.08 (0.88–1.32)	0.470
SIMD 4-5 (baseline)	1.02 (0.86–1.21)	0.837
Cardiovascular disease	1.32 (1.13–1.53)	<0.001
Cancer	1.01 (0.84–1.23)	0.903
Depression	1.56 (1.34–1.83)	<0.001
Diabetes mellitus	0.97 (0.81–1.18)	0.778
Chronic respiratory disease	1.16 (0.98–1.37)	0.090
eGFR creatinine	1.00 (0.99–1.01)	0.845
Gabapentinoid ever use	1.09 (0.75–1.60)	0.645
Opioid ever use	1.03 (0.88–1.20)	0.750
Cumulative 90d gabapentinoid exposure	0.91 (0.80–1.04)	0.155

Based on n=21,440 non-prevalent opioid or gabapentinoid users at baseline without missing SIMD. GEE logistic model including the following terms: cumulative 90 day gabapentinoid use (linear), duration of CKD diagnosis (years, centred, linear), current age (years, centred, linear), age x CKD duration interaction, eGFRcr (linear), cardiovascular disease, cancer, depression, respiratory disease, diabetes, ever prior opioid use, ever prior gabapentinoid use and baseline sex (fixed) and socioeconomic status (fixed). Estimates shown are odds ratios with Wald 95% CIs and p-values. Each variable is time-updated unless otherwise specified.

Supplemental Table S15: Marginal risk of death and odd ratios by 90-day cumulative lipid-lowering therapy

90-day continuous lipid-lowering therapy	Risk of death (95% CI)	Odds Ratio (95% CI)	P-value
0	0.4 (0.3–0.4)	1.00 (-)	NA
1	0.4 (0.3–0.4)	0.98 (0.97–0.99)	< 0.001
2	0.3 (0.3–0.4)	0.96 (0.94–0.97)	< 0.001
3	0.3 (0.3–0.3)	0.93 (0.91–0.96)	< 0.001
4	0.3 (0.3–0.3)	0.91 (0.88–0.95)	< 0.001
5	0.3 (0.3–0.3)	0.89 (0.86–0.93)	< 0.001
6	0.3 (0.3–0.3)	0.87 (0.83–0.92)	< 0.001

Based on n=21,440 non-prevalent opioid or gabapentinoid users at baseline without missing SIMD. Probability of death during the 28-day time interval having survived to the start of that interval derived from a GEE logistic model including the following terms: cumulative 90 day lipid-lowering therapy use (linear), cumulative 90 day lipid-lowering therapy use (quadratic), duration of CKD diagnosis (years, centred, linear), current age (years, centred, linear), age x CKD duration interaction, eGFRcr (linear), cardiovascular disease, cancer, depression, respiratory disease, diabetes, ever prior opioid use, ever prior gabapentinoid use and baseline sex (fixed) and socioeconomic status (fixed). OR are relative to an exposure of 0. Covariates time-updated unless otherwise specified. P values are Wald tests comparing each lipid-lowering therapy exposure level with the reference level derived from the fitted GEE model.

Supplemental Figure S1: Direct acyclic graph depicting the proposed relationship between chronic opioid exposure and mortality

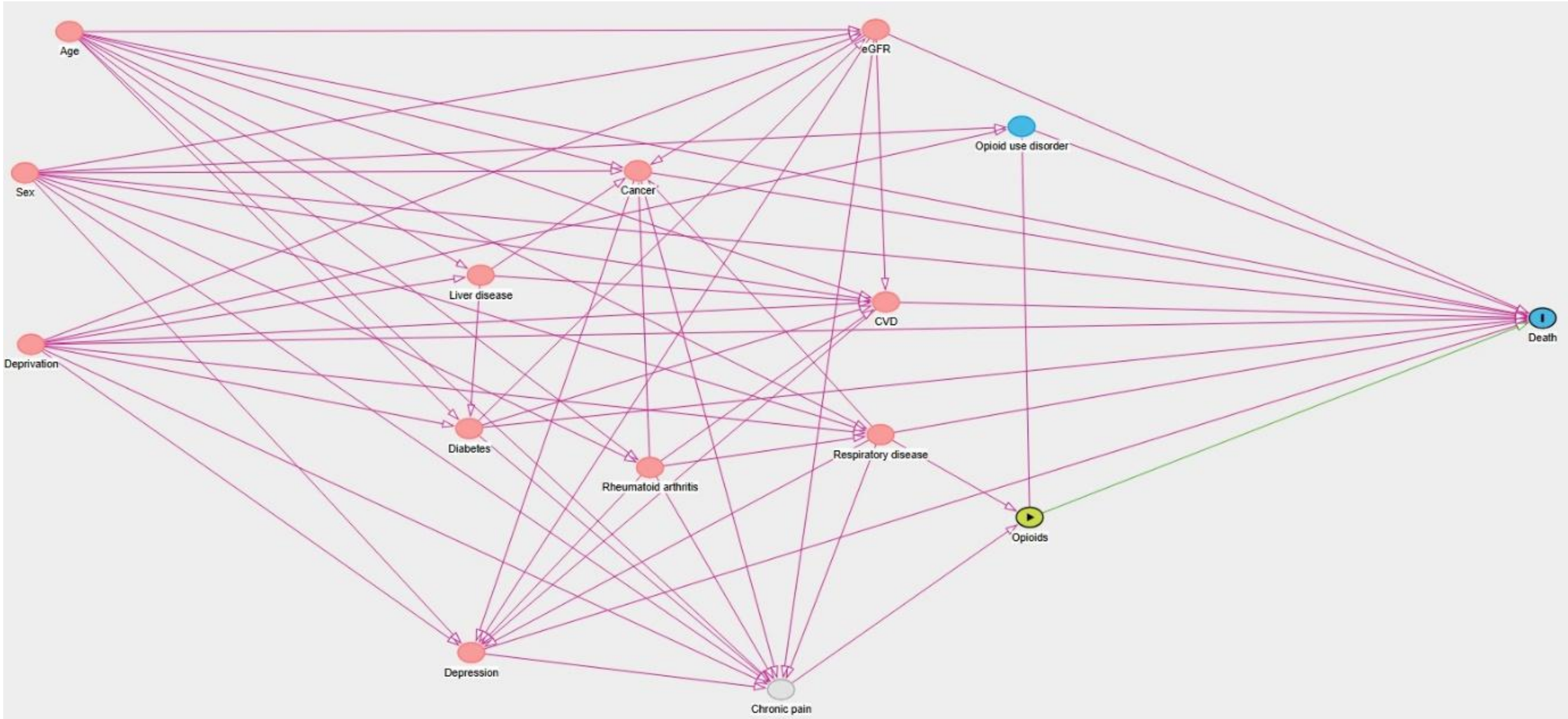

Supplemental Figure S2: Direct acyclic graph depicting the proposed relationship between chronic gabapentinoid exposure and mortality

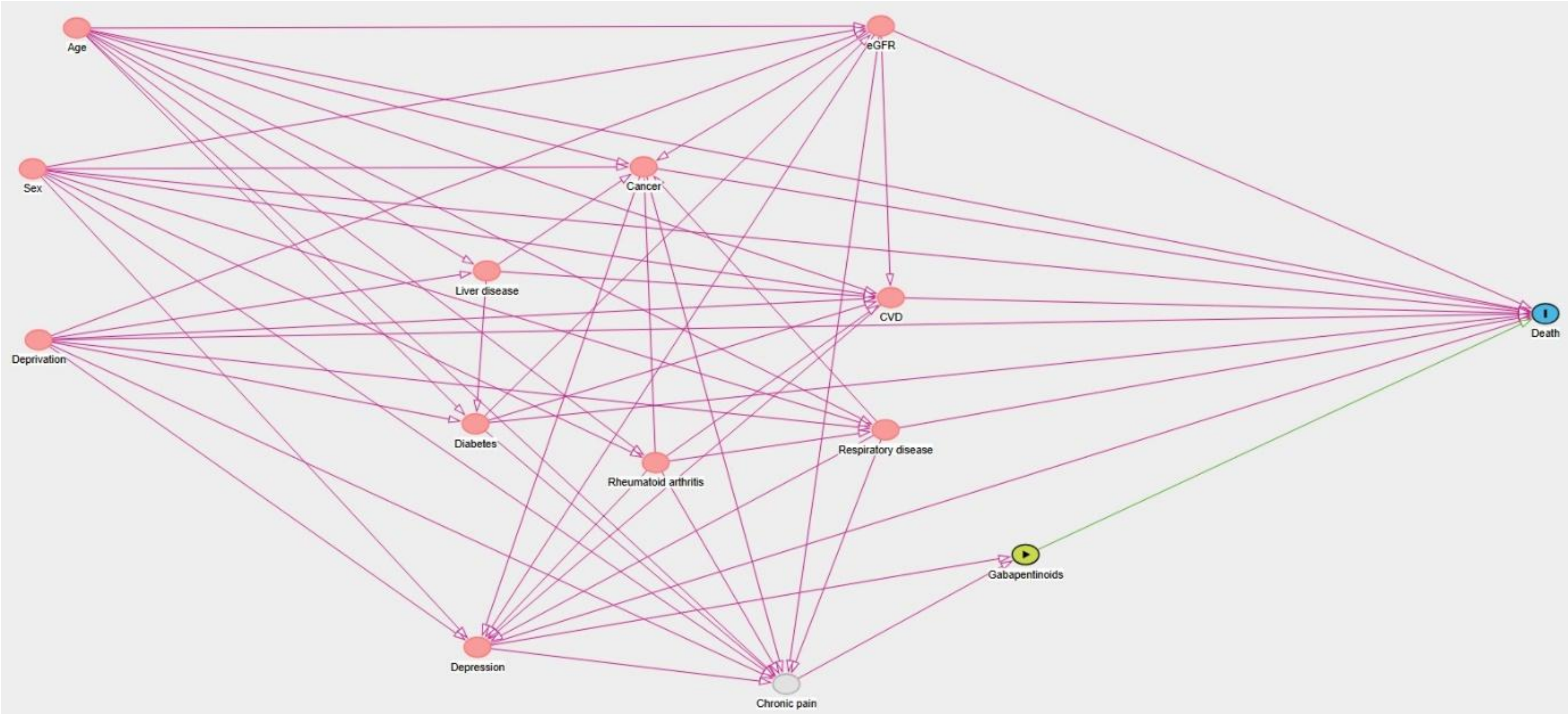

Supplemental Figure S3: Marginal risk of death by cumulative opioid exposure in females

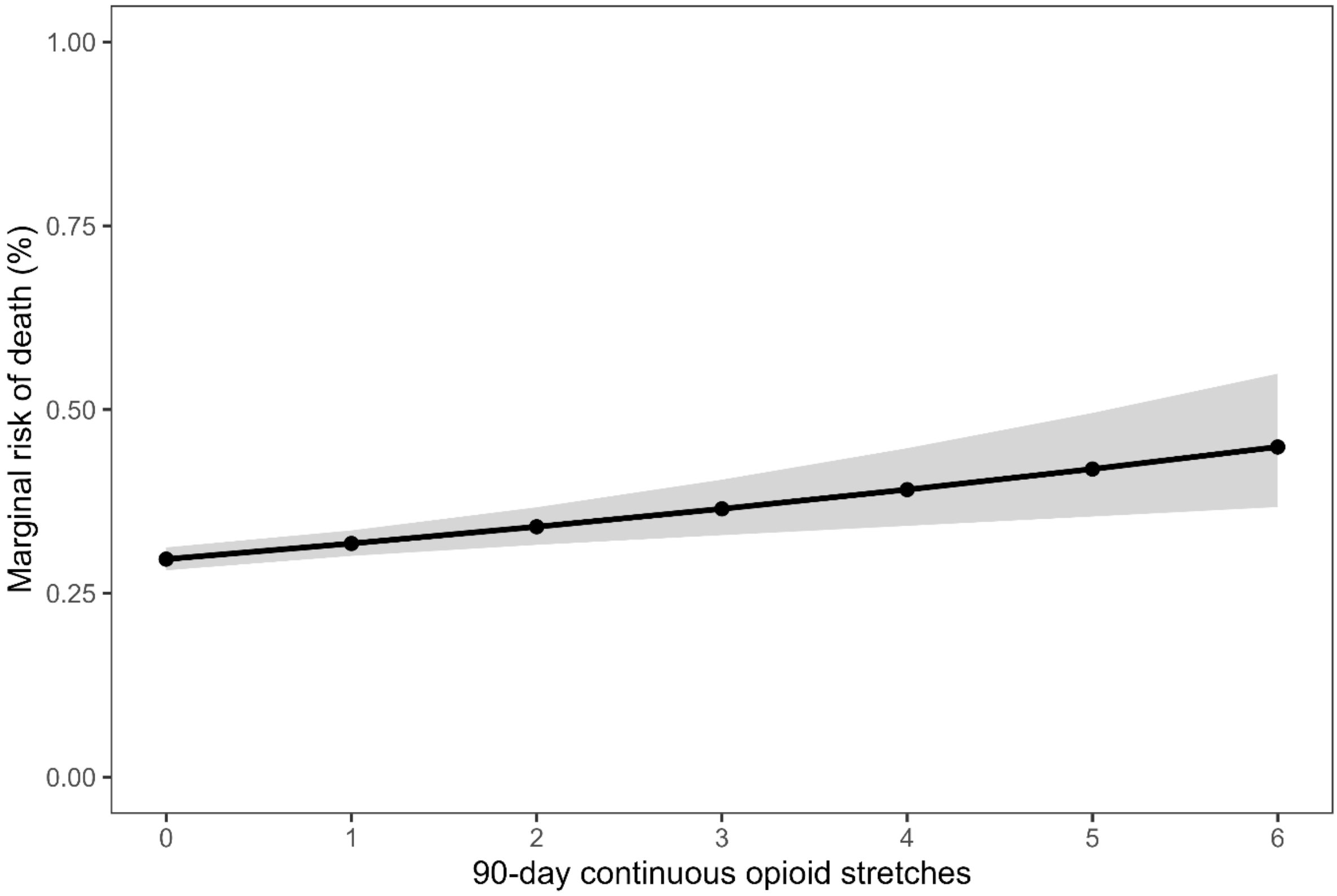

Supplemental Figure S4: Marginal risk of death by cumulative opioid exposure in males

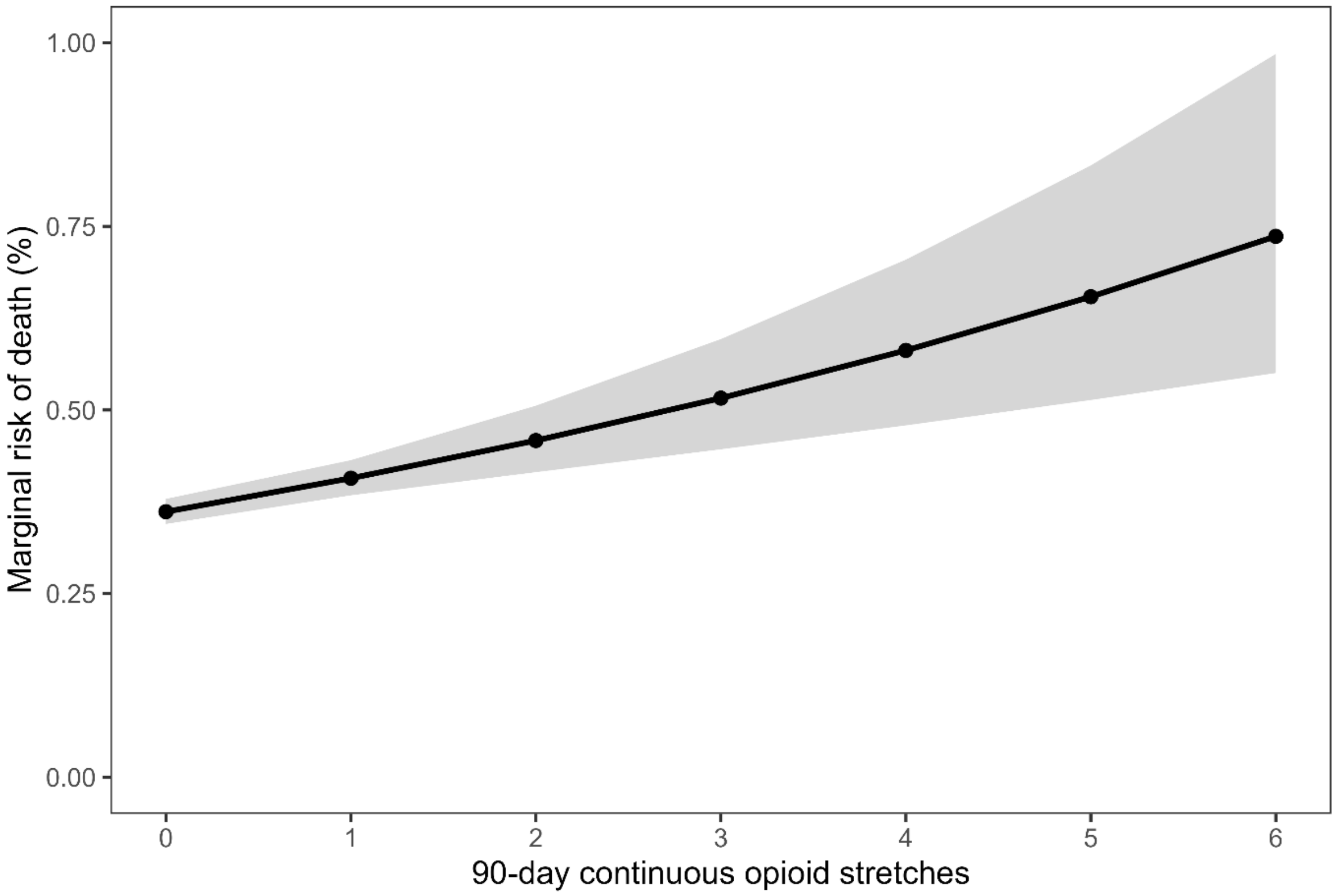

Supplemental Figure S5: Marginal rate of emergency hospitalisation by cumulative opioid exposure in females

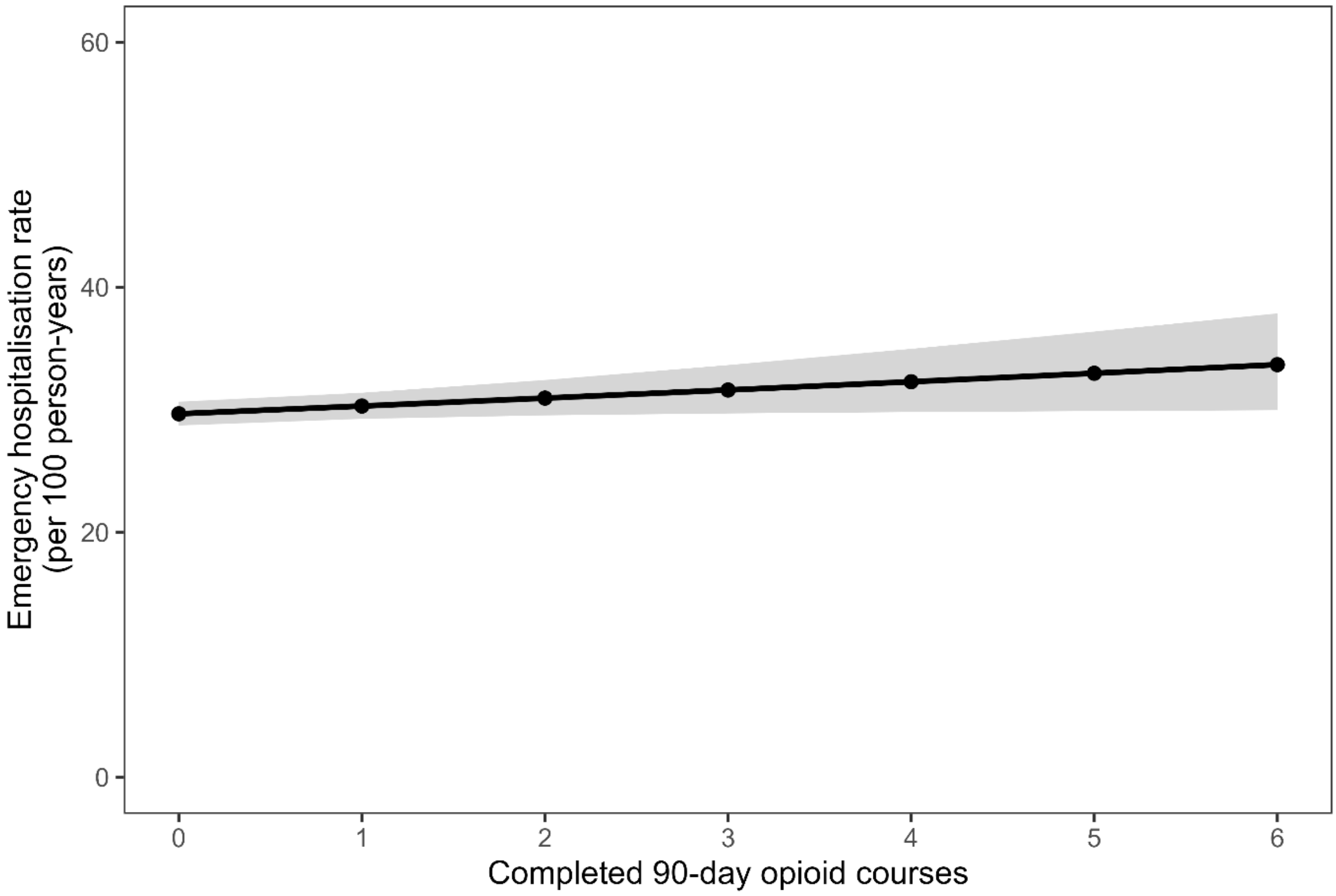

Supplemental Figure S6: Marginal rate of emergency hospitalisation by cumulative opioid exposure in males

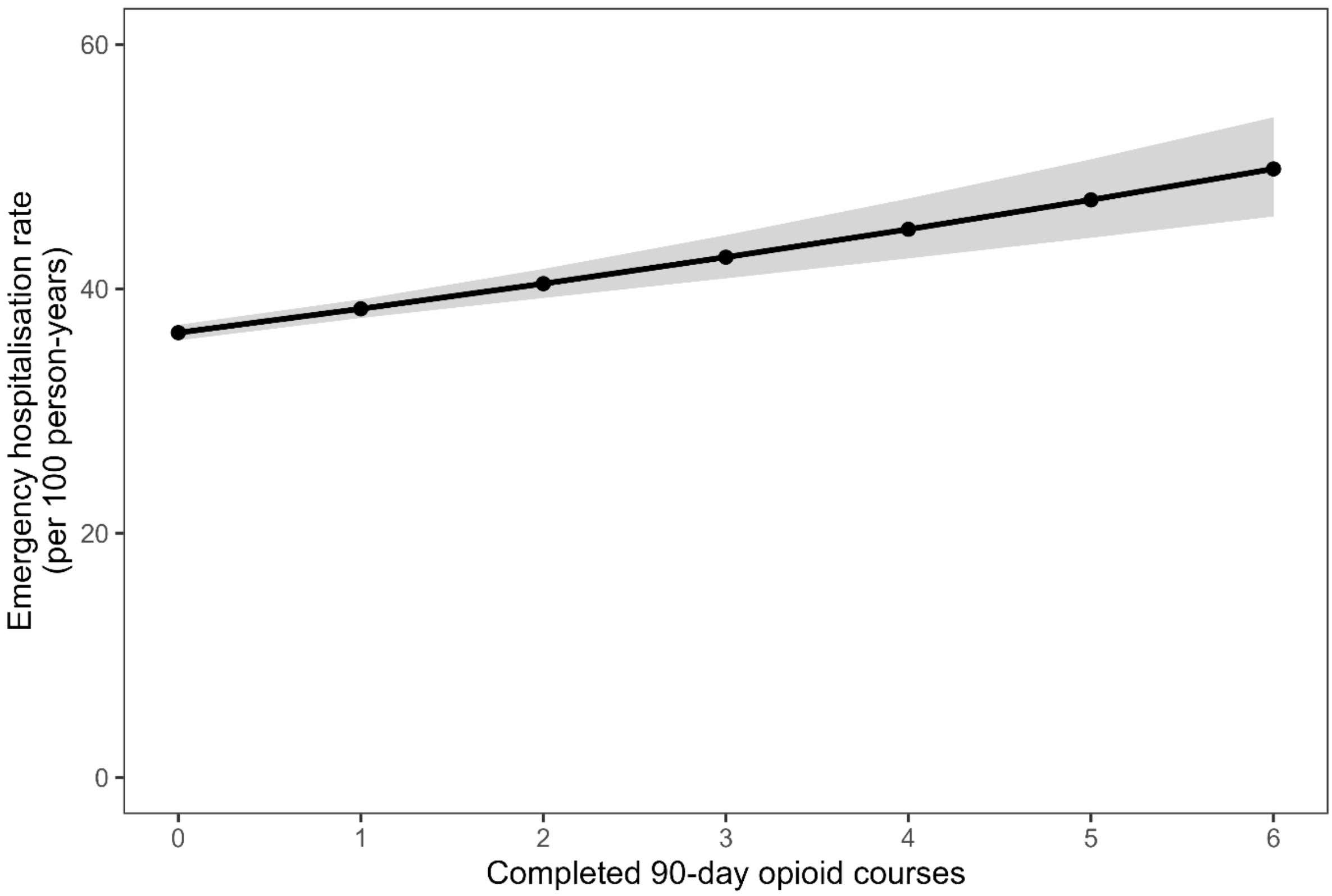

Supplementary References

S1. Public Health Scotland. CHI Number - Search the Data Dictionary 2021. Available from: https://publichealthscotland.scot/resources-and-tools/health-intelligence-and-data-management/national-data-catalogue/data-dictionary/search-the-data-dictionary/chi-number/.
S2. Health Informatics Centre. Demography 2022. Available from: https://discovery.dundee.ac.uk/en/datasets/demography.
S3. Public Health Scotland. General Acute Inpatient and Day Case - Scottish Morbidity Record (SMR01) 2024. Available from: https://publichealthscotland.scot/resources-and-tools/health-intelligence-and-data-management/national-data-catalogue/national-datasets/search-the-datasets/general-acute-inpatient-and-day-case-scottish-morbidity-record-smr01/.
S4. Gallacher PJ, Yeung D, Bell S, Shah ASV, Mills NL, Dhaun N. Kidney replacement therapy: trends in incidence, treatment, and outcomes of myocardial infarction and stroke in a nationwide Scottish study. European Heart Journal. 2024;45(15):1339–51.
S5. Public Health Scotland. Scottish Cancer Registry (SMR06). Available from: https://publichealthscotland.scot/resources-and-tools/health-intelligence-and-data-management/national-data-catalogue/national-datasets/search-the-datasets/scottish-cancer-registry-smr06/.
S6. National Records of Scotland. Scotland's Census 2022: Quality Assurance of Administrative Datasets 2022. Available from: https://www.scotlandscensus.gov.uk/media/orulpvdp/scotlands-census-2022-admin-data-2016-2022-quality-assurance-of-administrative-dataset.pdf.
S7. The University of Dundee. Health Informatics Centre - Dataset Inventory at HIC Available from: https://www.dundee.ac.uk/hic/data-service/dataset-inventory.
S8. Public Health Scotland. Prescribing Information System (PIS) 2024. Available from: https://publichealthscotland.scot/resources-and-tools/health-intelligence-and-data-management/national-data-catalogue/national-datasets/search-the-datasets/prescribing-information-system-pis/.
S9. Alvarez-Madrazo S, McTaggart S, Nangle C, Nicholson E, Bennie M. Data Resource Profile: The Scottish National Prescribing Information System (PIS). International Journal of Epidemiology. 2016;45(3):714–5f.
S10. The SCI-Diabetes Collaboration. SCI-Diabetes. Available from: https://www.sci-diabetes.scot.nhs.uk/.
S11. McGurnaghan SJ, Blackbourn LAK, Caparrotta TM, Mellor J, Barnett A, Collier A, et al. Cohort profile: the Scottish Diabetes Research Network national diabetes cohort - a population-based cohort of people with diabetes in Scotland. BMJ Open. 2022;12(10):e063046.
S12. Scottish National Audit Programme (SNAP. Scottish Renal Registry (SRR): Public Health Scotland. Available from: https://publichealthscotland.scot/resources-and-tools/health-strategy-and-outcomes/scottish-national-audit-programme-snap/scottish-renal-registry-srr/.
S13. Simpson K. The Scottish renal registry. Scottish medical journal. 1993;38(4):107–9.
S14. The Scottish Renal Association. The Scottish Renal Registry. Available from: https://www.srr.scot.nhs.uk/index.htm.
S15. National Records of Scotland. National Records of Scotland (NRS): Deaths Data. Available from: https://publichealthscotland.scot/resources-and-tools/health-intelligence-and-data-management/national-data-catalogue/national-datasets/search-the-datasets/national-records-of-scotland-nrs-deaths-data/#summary.
S16. National Records of Scotland. Registering a death. Available from: https://www.nrscotland.gov.uk/registration/registering-a-death/#:~:text=Deaths%20in%20Scotland%20should%20be%20registered%20within,be%20registered%20at%20any%20Registration%20office%20in.
S17. National Records of Scotland. Registering a death. Available from: https://www.nrscotland.gov.uk/registration/registering-a-

death/#:~:text=Deaths%20in%20Scotland%20should%20be%20registered%20within,be%20registered%20at%20any%20Registration%20office%20in.
S18. Mbizvo GK, Schnier C, Simpson CR, Duncan SE, Chin RFM. Validating the accuracy of administrative healthcare data identifying epilepsy in deceased adults: A Scottish data linkage study. Epilepsy Research. 2020;167:106462.
S19. Fernie CGM. Scotland already has world leading mortality review system. BMJ. 2019;364:l395.
S20. UK Kidney Association. Measurement of kidney function. Available from: https://www.ukkidney.org/health-professionals/information-resources/uk-eckd-guide/measurement-kidney-function.
S21. NICE. Chronic kidney disease: Assessment and management. London: National Institute for Health and Care Excellence. 2021.
S22. Torrance N, Mansoor R, Wang H, Gilbert S, Macfarlane GJ, Serpell M, et al. Association of opioid prescribing practices with chronic pain and benzodiazepine co-prescription: a primary care data linkage study. Br J Anaesth. 2018;120(6):1345–55.
S23. British National Formulary (online): BMJ and Pharmaceutical Press; 2023. Available from: https://www.medicinescomplete.com/.
S24. British National Forumulary. Frequently asked questions for the BNF and BNF for children. 2025. Available from: https://bnf.nice.org.uk/about/frequently-asked-questions-for-the-bnf-and-bnf-for-children-general/.
S25. NICE. Controlled drugs and drug dependence. Available from: https://bnf.nice.org.uk/medicines-guidance/controlled-drugs-and-drug-dependence/#:~:text=The%20Department%20of%20Health%20and,the%20medicine%20can%20be%20supplied.
S26. Glancy M, Palmateer N, Yeung A, Hickman M, Macleod J, Bishop J, et al. Risk of drug-related death associated with co-prescribing of gabapentinoids and Z-drugs among people receiving opioid-agonist treatment: A national retrospective cohort study. Psychiatry Res. 2024;339:116028.
S27. Safer Management of Controlled Drugs: A guide to good practice in the management of controlled drugs in primary care – Scotland (Version 3) 2023. Available from: https://www.communitypharmacy.scot.nhs.uk/media/7474/cdao-network-primary-care-good-practice-guide-april-23-002.pdf.
S28. Hsu CY, Liu KD, Yang J, Glidden DV, Tan TC, Pravoverov L, et al. Renin-Angiotensin System Blockade after Acute Kidney Injury (AKI) and Risk of Recurrent AKI. Clin J Am Soc Nephrol. 2020;15(1):26–34.
S29. Sikka R, Xia F, Aubert RE. Estimating medication persistency using administrative claims data. Am J Manag Care. 2005;11(7):449–57.
S30. Ziegler A, Vens M. Generalized estimating equations. Notes on the choice of the working correlation matrix. Methods Inf Med. 2010;49(5):421–5; discussion 6–32.
S31. Muller CJ, MacLehose RF. Estimating predicted probabilities from logistic regression: different methods correspond to different target populations. International Journal of Epidemiology. 2014;43(3):962–70.
S32. Textor J, Van der Zander B, Gilthorpe MS, Liśkiewicz M, Ellison GT. Robust causal inference using directed acyclic graphs: the R package ‘dagitty’. International journal of epidemiology. 2016 Dec 1;45(6):1887-94.
S33. Carrero JJ, Fu EL, Vestergaard SV, Jensen SK, Gasparini A, Mahalingasivam V, et al. Defining measures of kidney function in observational studies using routine health care data: methodological and reporting considerations. Kidney International. 2023;103(1):53–69.
S34. Lipsitch M, Tchetgen Tchetgen E, Cohen T. Negative controls: a tool for detecting confounding and bias in observational studies. Epidemiology. 2010;21(3):383–8.
S35. Public Health Scotland. Data Dictionary - Admission Type 2021. Available from: https://publichealthscotland.scot/resources-and-tools/health-intelligence-and-data-management/national-data-catalogue/data-dictionary/search-the-data-dictionary/admission-type/.

S36. Moore E, Munoz-Arroyo R, Schofield L, Radley A, Clark D, Isles C. Death within 1 year among emergency medical admissions to Scottish hospitals: incident cohort study. BMJ Open. 2018;8(6):e021432.
S37. Zhong H, Poeran J, Liu J, Wilson LA, Memtsoudis SG. Hip fracture characteristics and outcomes during COVID-19: a large retrospective national database review. British Journal of Anaesthesia. 2021;127(1):15–22.

RECORD-PE Checklist

The RECORD statement for pharmacoepidemiology (RECORD-PE) checklist of items, extended from the STROBE and RECORD statements, which should be reported in non-interventional pharmacoepidemiological studies using routinely collected health data

Item No	STROBE items	RECORD items	RECORD-PE items	Page No
**Title and abstract**				
1	(a) Indicate the study's design with a commonly used term in the title or the abstract.<br>(b) Provide in the abstract an informative and balanced summary of what was done and what was found.	1.1: The type of data used should be specified in the title or abstract. When possible, the name of the databases used should be included.<br>1.2: If applicable, the geographical region and timeframe within which the study took place should be reported in the title or abstract.<br>1.3: If linkage between databases was conducted for the study, this should be clearly stated in the title or abstract.	—	Title & abstract
**Introduction**				
Background rationale				
2	Explain the scientific background and rationale for the investigation being reported.	—	—	P4
Objectives				
3	State specific objectives, including any prespecified hypotheses.	—	—	P5
**Methods**				
Study design				
4	Present key elements of study design early in the paper.	—	4.a: Include details of the specific study design (and its features) and report the use of multiple designs if used.<br>4.b: The use of a diagram(s) is recommended to illustrate key aspects of the study design(s), including exposure, washout, lag and observation periods, and covariate definitions as relevant.	P5-P9<br><br>Suppl P6-14 + Suppl P28-29
Setting				

5	Describe the setting, locations, and relevant dates, including periods of recruitment, exposure, follow-up, and data collection.	—	—	P5-7
Participants				
6	(a) Cohort study—give the eligibility criteria, and the sources and methods of selection of participants. Describe methods of follow-up. Case-control study—give the eligibility criteria, and the sources and methods of case ascertainment and control selection. Give the rationale for the choice of cases and controls. Cross sectional study—give the eligibility criteria, and the sources and methods of selection of participants.<br><br>(b) Cohort study—for matched studies, give matching criteria and number of exposed and unexposed. Case-control study—for matched studies, give matching criteria and the number of controls per case.	6.1: The methods of study population selection (such as codes or algorithms used to identify participants) should be listed in detail. If this is not possible, an explanation should be provided.<br><br>6.2: Any validation studies of the codes or algorithms used to select the population should be referenced. If validation was conducted for this study and not published elsewhere, detailed methods and results should be provided.<br><br>6.3: If the study involved linkage of databases, consider use of a flow diagram or other graphical display to demonstrate the data linkage process, including the number of individuals with linked data at each stage.	6.1.a: Describe the study entry criteria and the order in which these criteria were applied to identify the study population. Specify whether only users with a specific indication were included and whether patients were allowed to enter the study population once or if multiple entries were permitted. See explanatory document for guidance related to matched designs.	P5-7, Figure 1 + Suppl P6-7
Variables				
7	Clearly define all outcomes, exposures, predictors, potential confounders, and effect modifiers. Give diagnostic criteria, if applicable.	7.1: A complete list of codes and algorithms used to classify exposures, outcomes, confounders, and effect modifiers should be provided. If these cannot be reported, an explanation should be provided.	7.1.a: Describe how the drug exposure definition was developed.<br><br>7.1.b: Specify the data sources from which drug exposure information for individuals was obtained.<br><br>7.1.c: Describe the time window(s) during which an individual is considered exposed to the drug(s). The rationale for selecting a particular time window should be provided. The extent of potential left truncation or left censoring should be specified.<br><br>7.1.d: Justify how events are attributed to current, prior, ever, or cumulative drug exposure.	P5-9<br><br>Suppl P5, 7-10<br><br>Complete list of drug codes available upon request<br><br>(see<br><br>Suppl)

			7.1.e: When examining drug dose and risk attribution, describe how current, historical or time on therapy are considered.<br><br>7.1.f: Use of any comparator groups should be outlined and justified.<br><br>7.1.g: Outline the approach used to handle individuals with more than one relevant drug exposure during the study period.	
Data sources/measurement				
8	For each variable of interest, give sources of data and details of methods of assessment (measurement). Describe comparability of assessment methods if there is more than one group.	—	8.a: Describe the healthcare system and mechanisms for generating the drug exposure records. Specify the care setting in which the drug(s) of interest was prescribed.	P5-6 & Supp P4-6
Bias				
9	Describe any efforts to address potential sources of bias.	—	—	P5-9 & Supp P8-11 & P28-29
Study size				
10	Explain how the study size was arrived at.	—	—	P5-6, Figure 1 & Supp P6-7
Quantitative variables				
11	Explain how quantitative variables were handled in the analyses. If applicable, describe which groupings were chosen, and why.	—	—	P6-9
Statistical methods				
12	(a) Describe all statistical methods, including those used to control for confounding.<br><br>(b) Describe any methods used to examine subgroups and interactions.<br><br>(c) Explain how missing data were addressed.<br><br>(d) Cohort study—if applicable, explain how loss to follow-up was addressed. Case-control study—if applicable, explain how matching	—	12.1.a: Describe the methods used to evaluate whether the assumptions have been met.<br><br>12.1.b: Describe and justify the use of multiple designs, design features, or analytical approaches.	P6-9 & Suppl P8-11

	of cases and controls was addressed. Cross sectional study—if applicable, describe analytical methods taking account of sampling strategy.<br><br>(e) Describe any sensitivity analyses.			
Data access and cleaning methods				
12	—	12.1: Authors should describe the extent to which the investigators had access to the database population used to create the study population.<br><br>12.2: Authors should provide information on the data cleaning methods used in the study.	—	Suppl P6
Linkage				
12	—	12.3: State whether the study included person level, institutional level, or other data linkage across two or more databases. The methods of linkage and methods of linkage quality evaluation should be provided.	—	P5
**Results**				
Participants				
13	(a) Report the numbers of individuals at each stage of the study (eg, numbers potentially eligible, examined for eligibility, confirmed eligible, included in the study, completing follow-up, and analysed).<br><br>(b) Give reasons for non-participation at each stage.<br><br>(c) Consider use of a flow diagram.	13.1: Describe in detail the selection of the individuals included in the study (that is, study population selection) including filtering based on data quality, data availability, and linkage. The selection of included individuals can be described in the text or by means of the study flow diagram.	—	Figure 1
Descriptive data				
14	(a) Give characteristics of study participants (eg, demographic, clinical, social) and information on exposures and potential confounders.<br><br>(b) Indicate the number of participants with missing data for each variable of interest.	—	—	Table 1, P9-1

	(c) Cohort study—summarise follow-up time (eg, average and total amount).			
Outcome data				
15	Cohort study—report numbers of outcome events or summary measures over time. Case-control study—report numbers in each exposure category, or summary measures of exposure. Cross sectional study—report numbers of outcome events or summary measures.	—	—	P9-11
Main results				
16	(a) Give unadjusted estimates and, if applicable, confounder adjusted estimates and their precision (eg, 95% confidence intervals). Make clear which confounders were adjusted for and why they were included.<br>(b) Report category boundaries when continuous variables are categorised.<br>(c) If relevant, consider translating estimates of relative risk into absolute risk for a meaningful time period.	—	—	Minimal adjusted models Suppl P18 + 20
Other analyses				
17	Report other analyses done—eg, analyses of subgroups and interactions, and sensitivity analyses.	—	—	P11-12 + Suppl P27
**Discussion**				
Key results				
18	Summarise key results with reference to study objectives.	—	—	P12
Limitations				
19	Discuss limitations of the study, taking into account sources of potential bias or imprecision. Discuss both direction and magnitude of any potential bias.	19.1: Discuss the implications of using data that were not created or collected to answer the specific research question(s). Include discussion of misclassification bias, unmeasured confounding, missing data, and changing eligibility over	19.1.a: Describe the degree to which the chosen database(s) adequately captures the drug exposure(s) of interest.	P13, Suppl P4-6

		time, as they pertain to the study being reported.		
Interpretation				
20	Give a cautious overall interpretation of results considering objectives, limitations, multiplicity of analyses, results from similar studies, and other relevant evidence.	—	20.a: Discuss the potential for confounding by indication, contraindication or disease severity or selection bias (healthy adherer/sick stopper) as alternative explanations for the study findings when relevant. **[A: Original text indicated this item was RECORD (ie, not RECORD-PE)?]**	P13-14
Generalisability				
21	Discuss the generalisability (external validity) of the study results.	—	—	P13
**Other information**				
Funding				
22	Give the source of funding and the role of the funders for the present study and, if applicable, for the original study on which the present article is based.	—	—	P16
Accessibility of protocol, raw data, and programming code				
22	—	22.1: Authors should provide information on how to access any supplemental information such as the study protocol, raw data, or programming code.	—	P17-18

RECORD=reporting of studies conducted using observational routinely collected data; RECORD-PE=RECORD for pharmacoepidemiological research; STROBE=strengthening the reporting of observational studies in epidemiology.

*REFERENCE: Langan SM, Schmidt S, Wing K, Ehrenstein V, Nicholls S, Filion K, Klungel O, Petersen I, Sorensen H, Guttmann A, Harron K, Hemkens L, Moher D, Schneeweiss S, Smeeth L, Sturkenboom M, von Elm E, Wang S, Benchimol EI. The REporting of studies Conducted using Observational Routinely-collected health Data (RECORD) Statement for Pharmacoepidemiology (RECORD-PE). *BMJ* 2018; 363: k3532.
